# Improving the use of plant genetic resources to sustain breeding programs’ efficiency

**DOI:** 10.1073/pnas.2205780119

**Published:** 2023-03-27

**Authors:** Dimitri Sanchez, Sarah Ben Sadoun, Tristan Mary-Huard, Antoine Allier, Laurence Moreau, Alain Charcosset

**Affiliations:** ^a^Université Paris-Saclay, Institut National de Recherche pour l’Agriculture, l’Alimentation et l’Environnement (INRAE), Centre National de la Recherche Scientifique (CNRS), AgroParisTech, Génétique Quantitative et Evolution (GQE) Le Moulon, 91190, Gif sur Yvette, France; ^b^Université Paris-Saclay, AgroParisTech, Institut National de la Recherche pour l'Agriculture, l'Alimentation et l'Environnement (INRAE), UMR Mathématiques et Informatique Appliquées (MIA) Paris-Saclay, 91120, Palaiseau, France; ^c^Syngenta, Saint-Sauveur 31790, France

**Keywords:** genetic diversity, bridging, breeding programs, optimal cross selection, genomic selection

## Abstract

Genetic progress of crop plants is required to face human population growth and guarantee production stability in increasingly unstable environmental conditions. Breeding is accompanied by a loss in genetic diversity, which hinders sustainable genetic gain. Methodologies based on molecular marker information have been developed to manage diversity and proved effective in increasing long-term genetic gain. However, with realistic plant breeding population sizes, diversity depletion in closed programs appears ineluctable, calling for the introduction of relevant diversity donors. Although maintained with significant efforts, genetic resource collections remain underutilized, due to a large performance gap with elite germplasm. Bridging populations created by crossing genetic resources to elite lines prior to introduction into elite programs can manage this gap efficiently. To improve this strategy, we explored with simulations different genomic prediction and genetic diversity management options for a global program involving a bridging and an elite component. We analyzed the dynamics of quantitative trait loci fixation and followed the fate of allele donors after their introduction into the breeding program. Allocating 25% of total experimental resources to create a bridging component appears highly beneficial. We showed that potential diversity donors should be selected based on their phenotype rather than genomic predictions calibrated with the ongoing breeding program. We recommend incorporating improved donors into the elite program using a global calibration of the genomic prediction model and optimal cross selection maintaining a constant diversity. These approaches use efficiently genetic resources to sustain genetic gain and maintain neutral diversity, improving the flexibility to address future breeding objectives.

## Significance

The increase and stability of crop production call for further genetic progress. To maintain it, breeders need to preserve genetic diversity in their programs, although it is depleted by the selection. During the last century, large collections of genetic resources have been organized, representing significant diversity. However, using them for variety development has remained an unsolved problem due to their performance gap with the elite varieties. Advances in genotyping and statistical methods now allow their efficient use through genomic prediction-based bridging schemes and diversity monitoring. Our study provides a demonstration of a suite of new tools incorporated into new breeding schemes to effectively resurrect donor accessions, enabling them to contribute to the improvement of quantitative traits in elite populations.

Modern plant selection can be traced back to the 19th century with pioneering work by the de Vilmorin family (1). They initiated the switch from single plant selection to that of families, i.e., genealogical selection. This led to a significant gain in heritability, one key driver of genetic gain in the breeder’s equation (2). It cleared the way for numerous methodological developments up to recent advances in genomic prediction. Numerous selection cycles in main annual crops have led to high genetic progress, which has contributed from 50 to 75% of productivity gains (3). Nevertheless, these advances have been accompanied by a loss of genetic diversity available for the farmers and the breeders (4–6). This may cause production instabilities in areas with adverse abiotic and biotic environmental conditions. It also may slow down genetic gain in breeding programs (2). In addition, several studies demonstrated that in the absence of specific constraints on diversity maintenance, recent selection methods such as genomic selection (GS) could accelerate the loss of genetic diversity by selecting highly related individuals (7–9). Diversity is also key to the evolution of breeding programs toward new selection objectives, such as those needed to anticipate climate change (10–12). Therefore, a suitable management of genetic diversity is needed. Recent advances in genotyping and statistical genetics methods offer new possibilities to achieve this objective.

Genetic diversity depletion within breeding populations can be slowed down using specific constraints. Methodologies to do so emerged initially in animal species for which the breeding population is directly used in production. In this case, excessive loss of diversity reduces production due to inbreeding depression, in addition to lowering additive genetic variance and therefore long-term genetic gain. A first approach is to impose restrictions on the number of progeny selected from any family (13). A more efficient one is to consider relatedness between the selected individuals and their contribution to the next generation (14, 15). These elements are incorporated in the optimized cross selection (OCS) index definition. OCS enables to select lists of parental crosses maximizing the mean expected breeding values of the progeny while constraining diversity to be above a predetermined decline trajectory. This was applied first using pedigree inferred relatedness and later extended to genotypic data. It has been shown that this approach can increase long-term genetic gain, with only minor short-term penalty (16, 17). Recently, Allier et al. (18) refined OCS in the context of GS schemes by developing the usefulness criterion parental contribution (UCPC). UCPC evaluates the interest of a list of crosses based on the expected genetic gain [UC (19)] and parental contributions (PC) of the selected individuals in the next generation. Simulations showed that the use of UCPC increased both short- and long-term genetic gains compared to OCS (20). In addition, other criteria were developed, such as the optimal haploid value (21) or the optimal population value (22), which are based on the selection of crosses or of a population, respectively, predicted to give the best doubled haploid progeny. Compared to GS without constraints on diversity, these aforementioned approaches preserve substantially more genetic diversity in the population by limiting the loss of low-frequency variants. However, with realistic plant breeding population sizes, diversity erosion in closed programs appears ineluctable in the long term.

Sustaining the efficiency of plant breeding programs therefore necessitates an appropriate diversity enrichment strategy. This strategy must i) identify relevant donors, i.e., individuals carrying favorable alleles not yet, or no longer, present in the breeding population and ii) incorporate these alleles into the elite population. Diversity sources in plant breeding can be categorized according to their readiness for use in an elite program. Two main factors impact this use: their “eliteness” and their environmental adaptation (*SI Appendix*, Fig. S1). Current competitor materials adapted to the target environment are the least challenging for incorporation. The accessibility of such resources depends on regulation rules. Varieties under protection by the International Union for the Protection of New Varieties of Plants (UPOV) are accessible for breeding, which guarantees a straightforward use for inbred varieties (*SI Appendix*, Fig. S1). Patents and Plant Variety Protection (PVP) delay the access to competitor materials until patent expiration, giving to the owner a substantial advantage. Incorporation of UPOV-protected and ex-PVP materials has become a routine procedure in breeding programs. In contrast, the inclusion of landraces and lines from collections of patrimonial materials (23) remains challenging.

The use of the diversity collections in breeding has long been limited by the genetic gap in performance with the elite programs. These collections were designated as “seed morgues” by Goodman in the 1980s (24). Nevertheless, successful introductions of favorable alleles originated from these resources were recorded for monogenic and oligogenic traits (25), and efficient marker-assisted backcross or gene pyramiding approaches have been proposed to facilitate them (26–29). Diversity enrichment strategies adapted to quantitative traits are more complex due to the polygenic nature of the traits but have also received long-term attention. Pioneering methodological work aiming at detecting useful individuals to complement elite materials was conducted by Dudley (30, 31). Cramer and Kannenberg (32) proposed a reshaped breeding program to transfer diversity from donors with an overall poor agronomic level to an elite breeding program through intermediate populations. These questions are currently renewed in the GS era (33, 34). Marker effects estimated using a genomic model calibrated with a suitable training population can help identify donor individuals that best complement an elite recipient population. Allier et al. (35) evaluated a criterion (H) using a sliding window along the genome to identify chromosomal regions where a donor outperforms the best individuals in the elite population. These regions can then be considered to target specific recombination events in the progeny of a donor × recipient cross (36). Regarding the incorporation step, Allier et al. (37) highlighted the benefit of introducing a bridging component in the selection scheme, i.e., selecting within the progeny of donor x elite crosses before introduction in the elite component. This appears as mandatory for the successful use of low-performance genetic resources, as they otherwise get introduced in the elite pool to sustain diversity, but with no chance of entering the pedigree of released elite varieties. This was recently confirmed by Vanavermaete et al. (38) and Breider et al. (39), who extended this idea by introducing a multicycle bridging component between the genetic resource component and the elite program. These studies showed a large long-term benefit of introducing genetic diversity, even from low-performing genetic resource collections, over closed elite schemes with an optimized diversity trajectory management.

**Fig. 1.**
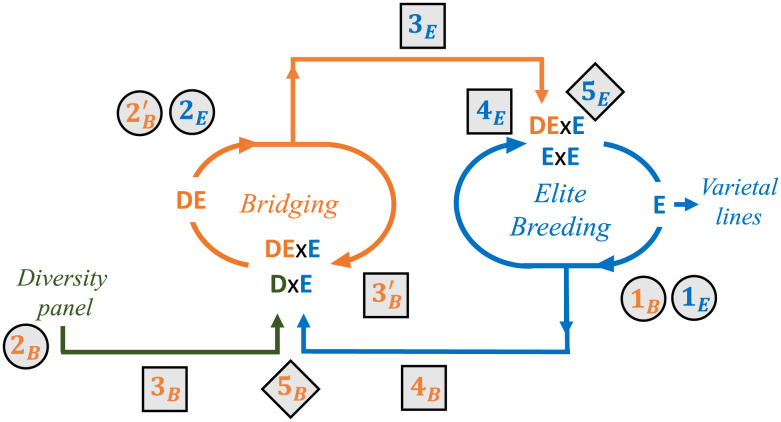
Global scheme for evaluated strategies. From left to right, donor detection from a diversity panel (green), donor improvement in a bridging breeding component (orange), and elite breeding phase to select new varieties (blue). Numbers indicate decisions to be taken at each selection cycle in the bridging (B) and elite (E) components: 1) Elite parent candidate evaluation, 2 and 2’) nonelite parent candidate evaluation, 3 and 3’) preselection of nonelite parent candidates, 4) preselection of elite parent candidates, and 5) cross list selection. The types of decisions are recalled by the shade: parent evaluation (circle), parent preselection (square), and cross selection (diamond).

The objective of our study is to further improve the use of genetic resource collection materials (classes + and ++ in *SI Appendix*, Fig. S1) in variety development, in a context where their overall gap in performance with elite materials increases over time. To do so, we consider as a general framework the scheme described in Fig. 1. We used the recent generic public simulator MoBPS [Modular Breeding Program Simulator (40)] to evaluate how the global efficiency of the program is affected by diversity management, genetic resources donor choice, genomic models used to select parental individuals and crosses of the bridging and elite components, with a hint in the allocation of resources to the different steps. We also analyzed the fate of donor alleles introduced in the program, the dynamics of diversity at neutral markers, and loci under selection. Our results emphasize the interest of phenotyping genetic resource collections and that of the bridging step to select donors which have the potential to unleash useful variation. They show that allocating 25% of total experimental resources to upstream bridging efforts increases mid- and long-term genetic gain and makes it possible to maintain diversity at currently neutral genomic regions, thus guaranteeing more flexibility to breed for new traits in a changing climatic and societal context.

## Results

### Advantages of Managing Diversity in a Closed Breeding Program.

We first evaluated as a benchmark a closed breeding program (i.e., elite population in Fig. 1) using different options to define the cross list. The first three are based on parental performances (phenotypic or predicted by a GS model) or the cross usefulness criterion (UC) which considers also the expected variance in progeny. The last two (OCS and UCPC-based OCS) use optimal cross selection methods to control the loss of genetic diversity at neutral markers (He) by imposing a decline trajectory. We selected 20 crosses each year and generated 80 progenies per cross.

As expected, OCS and UCPC-based OCS with a controlled decline in diversity led to the highest long-term genetic gain, with only a short-term penalty. (*SI Appendix*, Fig. S2 and Table S1). After 60 generations, all strategies led to a low level of genetic diversity. Note that attempting to maintain the elite diversity constant was not advantageous in the short term and it was too stringent so that long-term UCPC parental selection failed. In the following, we introduced external donors to maintain diversity. UCPC-based OCS with a controlled decline in diversity in a closed program was considered as a benchmark to evaluate the benefit of different open breeding strategies

### Usefulness of Donor Introductions using Bridging Strategies. Impact of GS Model Calibration and Diversity Management.

**Fig. 2.**
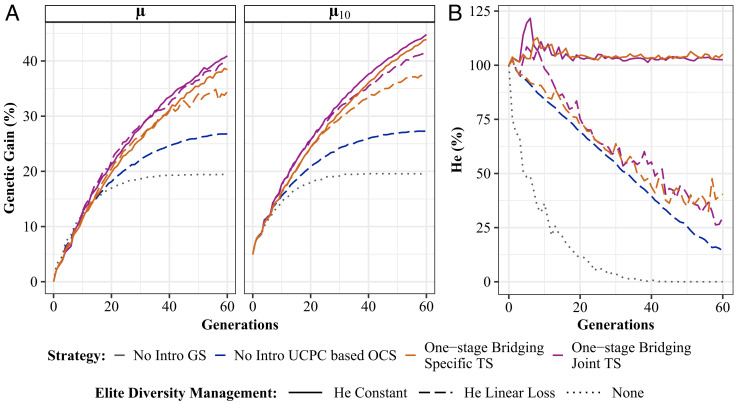
Evaluation of the impact of one-stage bridging strategies under different options for the GS model calibration and the elite diversity management. (*A*) Mean genetic gain for elite population (μ) and for the 10 best individuals ($\mu_{10}$) after the burn-in phase. (*B*) Elite neutral Nei diversity (He) in percentage of the neutral Nei diversity at the end of the burn-in phase. “No intro”: closed breeding program. “Specific TS” and “Joint TS”: choice of GS calibration model. “He Constant” and “He Linear Loss”: targeted elite diversity trajectory.

We centered our study on the introduction of diversity from gene bank collections, using different strategies listed in *SI Appendix*, Table S2. We first kept the same total breeding resources as for the closed program and attributed 25% (5 crosses per year) to a one-stage bridging component and 75% (15 crosses per year) to the elite component. This allocation of experimental resources is referred as $15C_{E}+5C_{B}$ ($C_{E}$: elite crosses, $C_{B}$: bridging crosses). All examined strategies surpassed the closed benchmark (UCPC) after 35 generations on the genetic gain (μ) and slightly earlier on the genetic gain at the commercial level ($\mu_{10}$: mean of the 10 most performing elite DH lines at a given generation, which mimics the mean value of released varieties from the elite program, Fig. 2). We did not notice any penalty on $\mu_{10}$ in previous generations. A joint GS model calibration on both bridging and elite components (Joint TS) appeared superior to using specific calibration (Specific TS) for each component (μ = 40.92% compared to μ = 38.42% after 60 generations, *SI Appendix*, Table S3). We used a Joint TS for the rest of our study. Keeping a constant elite diversity (He constant) led to lower μ values than allowing a linear diversity decline (He linear loss) until the generations 30 to 35, whereas we observed the reverse later. Focusing on $\mu_{10}$, the “He constant” performed similarly as “He linear loss” until generations 30 to 35 and outperformed it later. Regarding diversity, we observed a burst in the first generations of introductions (Fig. 2*B*). Diversity then followed the targeted trajectory. In the case of a linear decline trajectory, it exceeded the targeted values in the long term.

### Benefits of a Multiple-Stage Bridging Component on Genetic Gain.

**Fig. 3.**
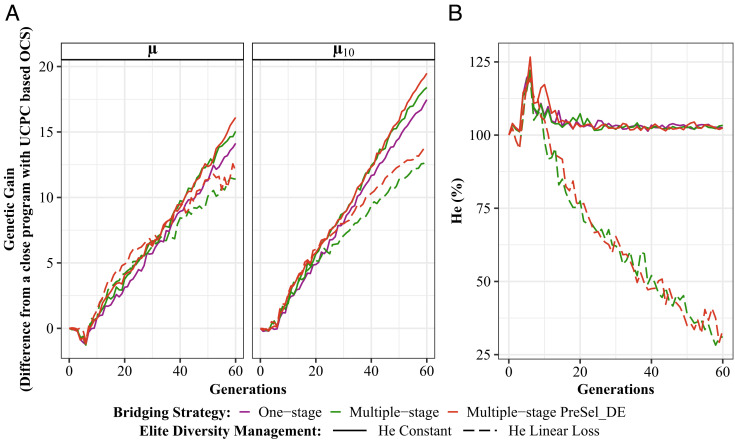
Comparison of one-stage and multiple-stage bridging strategies under different options for the elite diversity management. (*A*) Mean genetic gain for elite population (μ) and for the 10 best individuals ($\mu_{10}$) from the end of the burn-in phase reported as deviations from a closed program managed with UCPC-based OCS. (*B*) Elite neutral Nei diversity (He) in percentage of the neutral Nei diversity at the end of the burn-in phase. “One-stage”, “multiple-stage”, and “multiple-stage PreSel_DE”: bridging strategies, “PreSel_DE” indicates a preselection of DE progenies based on their performance. “He Constant” and “He Linear Loss”: targeted elite diversity trajectory.

To allow a progressive improvement of donor individuals before their incorporation into the elite component, we considered a multiple-stage bridging strategy, in which we allow crosses between bridging progeny and elite individuals (DExE) in addition to donor × elite crosses (Fig. 1). This was applied using all available bridging progenies or after intracross preselection to balance cross contribution. These alternatives were evaluated under both above-described diversity management regimes (Fig. 3 and *SI Appendix*, Table S4). We confirmed the superiority of the “He constant” for all options. Multiple-stage strategy with preselection of DE progeny appeared slightly superior than that without (μ = 46.80% and μ = 45.72%, respectively) and surpassed the one-stage strategy (μ = 44.79%) after 60 generations. Although beneficial, especially in the long term, multiple-stage strategy had a milder effect than diversity management.

### Advantage of Phenotype-Based Selection of Donors.

Donors were selected above based on UCPC of all possible DxE crosses (5B in Fig. 1), using the GS model calibrated with the Joint TS. We evaluated an alternative based on OCS and phenotypic evaluation of donors. This approach was implemented i) directly on the whole population of donors or ii) after preselection of 20 donors using criterion H to target original favorable chromosome segments superior to those of the elite component. Phenotype-based selection appeared beneficial for both μ and $\mu_{10}$ (*SI Appendix*, Fig. S3 and Table S5), but this advantage decreased for the most advanced generations. With the one-stage bridging strategy, at generation 60, $\mu_{10}$ = 43.79% and 45.67% when the donors are selected by GS or phenotyped, respectively. Preselecting donors based on the H criterion did not lead to a clear improvement. Note that at the beginning of the program, the phenotyping of donors reduced the advantage of the multistage bridging strategy.

### Effect of Breeding Resource Allocation.

We evaluated two additional experimental resource allocations between the bridging and elite component. We kept the same total number of individuals created each year as the initial allocation (1,600). For the first ($15C_{E}+10C_{B}$), we increased from 5 to 10 the cross number made at each generation in the bridging while reducing from 80 to 40 the progeny number per cross. For the second ($10C_{E}+10C_{B}$), we increased to 50% (10 crosses) rather than 25% (5 crosses) the experimental resources allocated to the bridging component, keeping 80 progenies by cross. $15C_{E}+10C_{B}$ allocation had a weak positive impact on μ and $\mu_{10}$ compared to the initial allocation, whereas $10C_{E}+10C_{B}$ allocation appeared prejudicial (*SI Appendix*, Fig. S4 and Table S5). The best for long-term genetic gain optimization was using the $15C_{E}+10C_{B}$ allocation combined with a preselection of donors based on the H criterion followed by phenotype-based selection.

### Dynamics of Diversity Evolution and Donor Contributions to the Elite Pool.

**Fig. 4.**
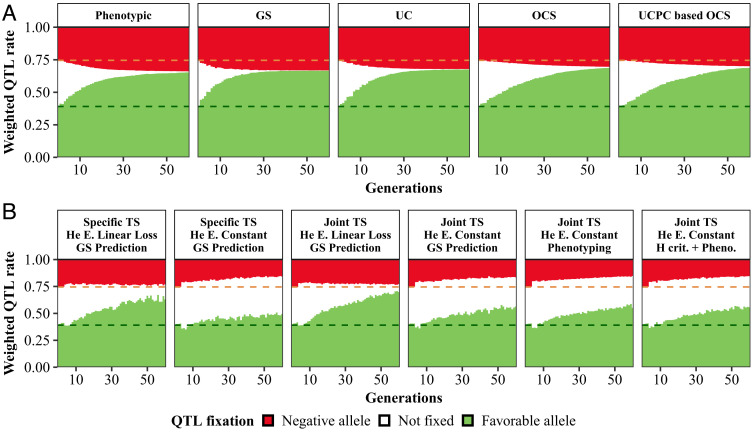
Evolution of fixation and polymorphism maintenance at QTLs in closed breeding programs (*A*) and in elite component of one-stage bridging programs (*B*). At each generation, QTLs are classified into 3 groups according to their status in elite population: fixed for the negative allele (red), fixed for the favorable allele (green), and not fixed (white). Weighted QTL rate of each category is computed as the sum of absolute effects of corresponding QTL, divided by the sum of absolute effects of all QTLs. Dashed lines indicate the values at the end of the burn-in phase. “Specific TS” and “Joint TS”: choice of GS calibration model. “He Constant” and “He Linear Loss”: targeted elite diversity trajectory. “GS prediction”, “Phenotyping”, and “H crit. + Pheno”: donors selection strategies. For bridging programs, 25% of breeding resources are allocated to the bridging component.

A closed elite program (Fig. 4*A* and *SI Appendix*, Table S6) led to the fixation or quasifixation of all quantitative trait loci (QTLs) at generation 60, including a substantial proportion of QTLs fixed for the unfavorable allele (${\mathrm{QTL}}^{-})$. Strategies with diversity management delayed the QTL fixation and led to a higher proportion of QTLs fixed for the favorable allele (${\mathrm{QTL}}^{+}$) than the classical GS strategy. For strategies involving donor introductions (Fig. 4*B* and *SI Appendix*, Table S7), the “He linear loss” diversity management allowed a rapid fixation of favorable alleles but the ${\mathrm{QTL}}^{-}$ proportion remained constant. Instead, the “He constant” allowed to efficiently decrease the ${\mathrm{QTL}}^{-}$ proportion over time. It also preserved a larger proportion of segregating QTLs. At generation 60 with a Joint TS and a one-stage bridging, 30.62% of QTLs was still segregating with this diversity management, whereas this number dropped to 9.02% with “He linear loss”.

**Fig. 5.**
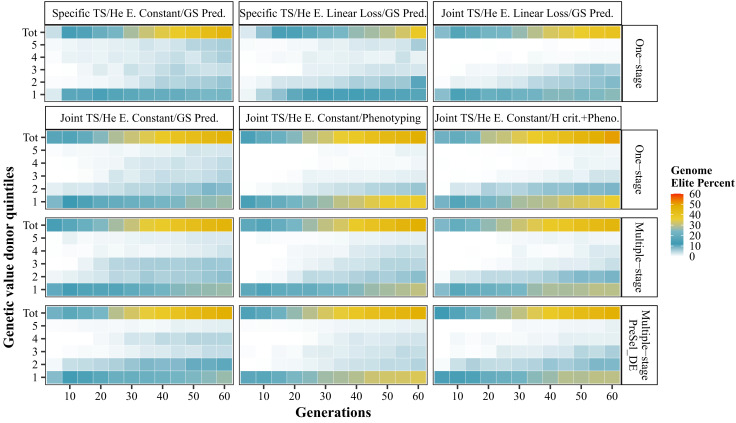
Relative contributions of introduced donors to the elite pool. Donors are ranked according to their genetic value quintiles, from 1 to 5 for the highest and lowest performing, respectively. Total contribution is also displayed (Tot). Contribution to elite genome is calculated based on pedigree information. “Specific TS” and “Joint TS”: choice of GS calibration model. “He Constant” and “He Linear Loss”: targeted elite diversity trajectory. “GS prediction”, “phenotyping”, and “H crit. + Pheno”: donors selection strategies. “One-stage”, “multiple-stage”, and “Loop NoPreSel_DE”: bridging strategies. For bridging programs, 25% of breeding resources are allocated to the bridging component.

In scenarios with introductions, the average proportion of donor genome in the elite population increased over time to reach between 37.4% and 56.7% at generation 60 (*SI Appendix*, Table S8). Using a Joint TS instead of a Specific TS reduced the number of different donors incorporated in the elite population ($N_{\mathrm{ID}})$ (28.4 instead of 45.6), but a higher proportion of them had progenies up to the generation 60. Selecting donors based on their phenotype had an even stronger effect: $N_{\mathrm{ID}}$ dropped but more than three-quarters of the introduced donors had contributed up to generation 60 (*SI Appendix*, Table S8). Phenotype-based selection of donors, and to a lesser extent the use of a Joint TS, selected first donors in the best true breeding value (TBV) quintile and then progressively donors with lower TBVs (Fig. 5).

## Discussion

### Limits of Closed Breeding Programs.

Maintaining genetic diversity in a breeding program is essential to guarantee sustainable genetic gain for targeted traits. Reduction in genotypic costs now allows to do so by implementing efficient molecular marker-based methods, such as those using optimal cross selection. We confirmed that they lead to a higher long-term genetic gain than selecting parents based on their phenotype or genomic prediction and intermating them randomly (37). The advantage of these methods comes from their ability to limit the involuntary fixation of negative alleles at QTLs due to drift or linkage with favorable alleles (Fig. 4). We developed optimization algorithms to facilitate their implementation. Nevertheless, our results and other long-term simulation studies (37, 41, 42) showed that, with realistic plant breeding settings, a genetic gain plateau is reached in all cases. Thus, introducing relevant sources of diversity is necessary to avoid it.

### Beneficial Introduction of Genetic Resources through Improved Bridging Schemes.

Diversity sources can be categorized according to their readiness for incorporation into an elite program (*SI Appendix*, Fig. S1). In this study, we focused on the use of adapted materials preserved in gene banks, created more than 20 y before the elite program was started. *SI Appendix*, Fig. S5 illustrates that this corresponds to a large genetic gap between donors and elite material, which increases over time as the elite program progresses. We show a clear benefit of enriching diversity using such genetic resources through a bridging component, i.e., by preselecting within the progeny of donor x elite crosses prior to their incorporation into the elite program. This confirms previous findings (37). This advantage can be obtained with no additional cost for programs that already have implemented GS by reallocating a moderate fraction of the elite program resources to the bridging component. This strategy comes with no or very limited penalty in the short term and a beneficial impact on genetic gain at commercial level after 10 generations with our simulation settings. This delay is expected to be reduced with a lower initial diversity level in the elite component and a higher donor richness in favorable alleles.

Using only 25% of experimental resources for implementing the bridging appeared more efficient than using 50%. For given resources allocated to the bridging program, we recommend preserving a substantial number of progeny per donor x elite cross rather than increasing the number of crosses. This is required to estimate accurately enough the variation generated by each cross to select valuable donors and also train appropriately the genomic prediction model for the newly introduced alleles.

### Identification and Preservation of Useful Variations.

Our results further lead to two important recommendations to ensure successful introduction.

*Preselection of genetic resources should be performed based on phenotypic data rather than based on the GS model trained on the breeding program.* This was true especially at the beginning of the program, when only a limited fraction of the genetic resource collection was integrated into the bridging program. It links to the well-known property of GS to favor individuals related to the training set (7–9) due to the impossibility for this approach to detect the interest of alleles not segregating within the training set (43). Phenotype-based selection of diversity donors reduced the number of donors entering the bridging component, but a higher proportion of these contributed to varietal release (*SI Appendix*, Table S8). This importance of phenotyping donors supports initiatives devoted to the phenotypic characterization of genetic resource collections, complemented by genomic prediction model training to facilitate prescreening (34, 44). This characterization should focus on performance (yield potential), tolerance to abiotic stresses and diseases, phenology, and also defaults like lodging to limit as much as possible later corrective efforts. This requires international coordination to have access to large trialing networks. Such initiatives, including public–private partnership are being deployed for several crops (45, 46) and must be generalized. A specific focus should be made on genotype x environment interaction to address production stability and adaptation to climate change, with a characterization of potential donors in diverse environmental conditions. Combination of field network and phenotyping platform with GS and ecophysological modeling (47) should prove instrumental in detecting genetic resources bringing new adaptive features.

Note that phenotype-based selection of donors hinders the possibility to predict the variance of donor × elite crosses. In this case, crosses can be selected based on their average performance and their contribution to the diversity inferred from genotypic data, using OCS algorithms. This approach proved efficient but may be improved by calibrating a specific GS model for donor selection based on the genetic resource panel itself. This option may be further improved by adding representative individuals from the elite germplasm and also individuals not selected (to represent the whole range of elite alleles) (48). Optimizing such strategies should be conducted using specific simulations which were beyond the scope of the present study.

*GS models calibrated with a same global training set (Joint TS) should be used for evaluating all types of candidate crosses.* With respect to genetic gain, this option appeared superior to selecting the crosses within the bridging and elite components with specific models (Specific TS). It also led to a higher incorporation rate of DE progeny in the elite germplasm (*SI Appendix*, Table S8). This suggests that one major interest of the bridging component of the program is to create and phenotype intermediate individuals between donors and elites that make it possible to evaluate contrasts between alleles of the elite pool and newly introduced alleles.

Despite an efficient phenotype-based selection of donors, we observed that only part of the donors introduced in the bridging component had a progeny selected for incorporation into the elite component (*SI Appendix*, Table S8). This might be explained by differences in the variability of the DE progeny depending on the donor. Such differences were observed in nested association mapping population in maize and were poorly predicted by the genetic distance between parents for traits with a complex genetic architecture (49, 50). GS-based prediction of variances would be highly beneficial but, as stated above, GS cannot anticipate the potential of donors with a low relatedness to the elite pool. One important aspect of the bridging crosses is thus to evaluate experimentally such variation, which necessitates sufficient progeny number for each donor x elite cross (40 or 80 in our settings).

### Advantage of Rapidly Recycling Improved Donor Progeny within a High Diversity Elite Component.

For donors with low performance, several cycles of crossing to the elite are needed to generate progeny competitive with elite lines. Pedigree examination showed that, with UCPC-based OCS, the elite diversity constraint allowed to preserve a part of introduced DE progeny and improve it by several crosses in the elite component (*SI Appendix*, Fig. S6). During this phase, crosses between DE progenies can also reveal complementarity between different donors. Vanavermaete et al. (38) showed an interest to have additional rounds of crosses to elite individuals for DE progenies within the bridging component. To understand the dynamics of the DE progeny improvement, we evaluated scenarios which combine the recycling of DE progenies in the bridging component and an elite diversity management. These scenarios did not lead to a substantial improvement of genetic gain in comparison with a one-stage bridging program despite a strong performance differentiation between elite and genetic resources (*SI Appendix*, Fig. S5). We observed that DE progenies were crossed to new E individuals essentially in the elite component, and their recycling in the bridging component was rarely used (*SI Appendix*, Fig. S6 and Table S9). An alternative diversity management with a weaker constraint on the elite diversity promoted the ExE crosses in the elite component at the expense of DExE crosses. It led to more frequent cycling of DE progeny in the bridging but without improving the final genetic gain (*SI Appendix*, Table S9). This confirms the impact of the diversity management on DE progeny improvement dynamics. Further investigations could be useful to find an optimal diversity management to ensure DE progeny improvement and maximize genetic gain in the elite component.

### Benefit of Maintaining a Global Diversity at Different Scales.

Maintaining diversity is crucial to rapidly adjust programs to changing breeding targets in response to climatic and societal changes. For open breeding programs, we showed that application of the UCPC-based OCS method can maintain diversity at neutral loci while increasing the frequency of the favorable alleles at QTLs (Fig. 4). Fixation of favorable alleles at QTLs is expected to lead to a loss of diversity at closely linked neutral markers, generating a tension between genetic gain and diversity maintenance. Our simulation settings involved 1,000 QTLs, so that most regions could not be considered “neutral”. Constant global diversity is therefore ensured by i) maximizing the diversity in regions with small cumulative effects for the trait of interest or ii) breaking LD in regions with larger effects so that a given favorable allele is carried by different haplotypes. These phenomena favor the occurrence of recombination events which can generate new haplotypic combinations at QTLs.

Our results underline the importance of the introduction of genetic diversity in individual breeding programs to ensure the release of original varieties and diversify the portfolio. Efforts to measure and monitor diversity of varieties deployed in a given agricultural landscape should be considered as a further step, using indices reviewed in Khoury et al. (4). Public policies that would encourage diversity maintenance at the landscape level are expected to have a positive impact on food security thanks to a better resilience to unpredictable climatic conditions. For example, considering genetic distance to existing varieties as a bonus in registration process may conduct breeders to consider explicitly the originality of released varieties as a target in their programs, providing a further incentive to use genetic resources collections.

### Conclusion and Future Work.

Our results support that the introduction of diversity from gene banks, which has long been questioned by breeders, can now be reconsidered using new tools and breeding strategies. They confirm the interest of the bridging strategies to introduce resources from collection materials. Our approach should be particularly useful to counterbalance the effect of genetic drift for breeding programs conducted with a small effective population size. This is the case of programs targeting market niches, which have limited resources and/or limited access to external elite resources, calling for incorporations from diversity collections. It also should be beneficial for larger breeding programs with restricted access to external elite germplasm or when programs of all competitors converge toward a same genetic pool with limited diversity (see *Discussion* above on diversity at the landscape level). More generally, we recommend performing a systematic assessment of the joint evolution of genetic gain, genetic variance, and genetic diversity, to detect situations, in which diversity erosion has a negative impact on genetic gain and calls for a reenrichment. (35) This approach should also help to estimate empirically the level of diversity to be targeted in the elite program.

Our results refine the roles of a bridging population. Beyond the reduction of the performance gap between diversity donors and the elite materials, which was its initial motivation, it contrasts the donor and elite alleles to calibrate efficiently GS models used to valorize the genetic variability. It also selects donors generating useful variation when crossed to the elite population. In our simulations, all candidates were phenotyped and GS models were recalibrated at each generation. A work to determine an optimal frequency of GS model recalibration would be interesting to possibly reduce phenotyping cost and accelerate breeding cycles.

Future work may also consider different trait simulation settings. Our single trait simulation setting can reflect a multitrait selection based on a linear combination but a more explicit multitrait simulation may be valuable (51). Also, we neglected the slowdown of the genetic variance erosion due to mutations and epistatic interactions (52–54). We focus here on the additivity that reflects well autogamous breeding or allogamous breeding conducted with a tester line. However alternative GS-based schemes are currently being developed in allogamous breeding (55, 56), and dominance should be considered to manage the complementarity between breeding populations, leading to specific constraints in the monitoring of genetic diversity. Our study could also be extended to test the adequacy of bridging strategies to more exotic material introductions with maladaptation calling for a preselection for specific traits. For landraces, the extraction of improved materials such as double haploid lines allows facilitating their evaluation and preselection (57).

Finally, new breeding technologies are increasingly used in breeding programs for targeted modifications of DNA guided by knowledge of key genes and favorable allele sequences. It has been demonstrated that editing few key genes permits de novo domestication of wild accessions (58, 59). While preserving their genomic originality, this brings such resources to a level suitable for introduction in a bridging program (level “+” in *SI Appendix*, Fig. S1). Gene editing can also be considered to create de novo allelic series for genes involved in agronomical interest traits (60). Introducing such materials among donors of the bridging component (Fig. 1) is an avenue to evaluate de novo and natural alleles for their exploitation in the elite compartment. We therefore hypothesize that rather than competing with each other, approaches presented in this study should be highly synergistic with new breeding technologies.

## Materials and Methods

### Genetic Material and Simulated Trait Architecture.

Simulations were conducted based on genotypic data of maize lines of the Amaizing dent diversity panel (43), which covers founder germplasm of dent commercial breeding. Lines were genotyped with single-nucleotide polymorphisms (SNPs) from the Illumina MaizeSNP50 BeadChip (61). QC filters (line call rate ≥ 0.8, marker call rate ≥ 0.9, line heterozygosity rate ≤ 0.1, and marker heterozygosity rate ≤ 0.15) lead to 338 lines and 41,495 high-quality SNPs. This panel was structured into three main groups: 57 Iodents, 82 Iowa Stiff Stalk, and 199 other dents.

Simulations were performed using R scripts (62) and the R package MoBPS (40). We simulated one quantitative trait influenced by 1000 biallelic QTLs sampled among the SNPs, with a minimal distance between two consecutive QTLs of 0.2 cM. Additive effects at QTLs were sampled from a centered Gaussian distribution with a variance of 0.05. We also sampled 2,000 SNPs as neutral markers. Genetic positions were determined using the dent consensus map (63).

### Initial Elite Program.

We simulated an elite breeding program (E) using the 57 Iodent lines as founders. Progeny was obtained using doubled-haploid (DH) technology. Recombination events along chromosomes were determined using a Poisson distribution, as suggested by the MoBPS user guide. We assumed 3 y to produce genotype and phenotype DH progeny. We considered overlapping generations with the selection of generation T parents from generations T-3, T-4, and T-5 to represent situations encountered in actual breeding programs. To initiate E progeny, 20 random founders were arbitrarily crossed with replacement during the first three generations, to create every year 10 families of 80 DH each. During  the next 17 generations, the 50 E progeny with the best phenotypic values from the generations T-3, T-4, and T-5 were selected and randomly intermated to create 20 biparental families of 80 DH each. Phenotypic values were inferred from the genotype at QTLs and an error variance corresponding to a trait heritability of 0.7 in the founder population, mimicking an usual multitrial phenotypic evaluation with a single replicate in 4 environments and a repeatability of 0.37. Starting from the initial elite program, different breeding strategies were conducted for 60 generations, including or not including diversity donor (D individuals) introductions using a bridging component. We replicated the procedure 10 times.

### Benchmark Implementation: Closed Elite Breeding Programs.

Closed breeding programs were implemented after the burn-in phase, with parental cross list selection guided by either i) the mean parental phenotypic values, ii) the mean parental GS predicted values (GEBV), iii) the cross UC, (19), iv) a classical OCS (see below, ref. 17), or v) a UCPC-based OCS. (see below, ref. 18). For the first option, 5% E progenies (i.e., 4 DH) with the highest phenotypic performance within each family of generations T-3, T-4, and T-5 were preselected as parents (i.e., 240 DH). The 20 biparental crosses with the best mean parental phenotypic values were selected to produce the next generation.

For other options, E progenies were genotyped for the 2,000 neutral markers and selection was based on a GBLUP model trained on generations T-3, T-4, and T-5 (i.e., 4,800 E progeny). To reduce computation time, genome-based estimated breeding values (GEBVs) were computed using the “direct approach” of MoPBS, which considers a fixed error variance. Marker effects were estimated by backsolving (64). We performed the same within family preselection as described above, using GEBVs instead of phenotypic values to determine potential parents. For the second and third options, we selected the 20 crosses with the best parental mean GEBVs and UC values, respectively. For a cross between lines j and k, UC was calculated as

[1] $$UC_{\mathrm{jk}}=\frac{GEBV_{j}+GEBV_{k}}{2}+ih\sigma_{\mathrm{jk}}$$

where i is the selection intensity (here i=2.06), h is the evaluation accuracy, and $\sigma_{\mathrm{jk}}$ is the expected genetic standard deviation in progeny. As in (18), we considered h=1 for sake of simplicity. $\sigma_{\mathrm{jk}}$ was estimated using estimated marker effects and linkage disequilibrium between markers inferred from the genetic map, as proposed by (65). In the last two options, further management of the genetic diversity was done using OCS methods described in *SI Appendix*, SI Text. For all options, 80 DH were derived for each cross (i.e., 1,600 DH per generation).

### One-Stage Bridging Implementation.

We simulated different breeding programs with donor introduction through a bridging component (Fig. 1), keeping resources constant (i.e., 1,600 DH per generation). Donor individuals came from the whole diversity panel simulating a static genetic resources collection. They were first crossed to elite lines (DxE crosses) to obtain bridging progeny (DE individuals). Part of DE individuals were then selected to be introduced into the elite component (DExE crosses). Three resource allocation scenarios were considered: i) a bridging population of 5 families of 80 DH (i.e., 400 DE progeny) and an elite population of 15 families of 80 DH (i.e., 1,200 E progeny), ii) a bridging population of 10 families of 40 DH (i.e., 400 DE progeny) and an elite population of 15 families of 80 DH (i.e., 1,200 E progeny), and iii) a bridging population of 10 families of 80 DH (i.e., 800 DE progeny) and an elite population of 10 families of 80 DH (i.e., 800 E progeny). We restrained E potential parents to the 5% E progeny per family (4 DH) with the highest GEBVs in generations T-3, T-4, and T-5 (i.e., 180 DH for i and ii vs. 120 DH for iii). DE potential parents for introduction in the elite program were selected from the DE progeny (i.e., 60 DH for i and ii vs. 120 DH for iii). For these preselection steps, GEBVs were estimated with two distinct GS models calibrated, respectively, on E progeny (i.e., 2,400 or 3,600 DH) and DE progeny (i.e., 2,400 or 1,200 DH). Elite crosses were selected using an UCPC-based OCS (*SI Appendix*, SI Text) with an elite GS model (“Specific TS” in *SI Appendix*, Table S2) or a GS model calibrated on both components (summing up to 4,800 DH—“Joint TS” in *SI Appendix*, Table S2). DxE bridging crosses were selected among all possible crosses between D (i.e., 368 lines) and preselected E progeny using a bridging specific GS model or the joint GS model. This selection was performed conditionally to the elite crosses from the same generation to complement the elite component by the bridging component (*SI Appendix*, SI Text). Note that calibrating bridging-specific or joint GS models required three generations after the burn-in phase, so that they could first be used at generation 24. Before this, the bridging cross selections were performed using the elite specific GS model.

### Multiple-Stage Bridging Implementation.

To implement the potential recycling of donors within the bridging component, we adapted previous settings to allow DExE crosses in addition to DxE crosses in bridging. Two options were considered to determine DE candidate parents: i) all DE progeny from generations T-3, T-4, or T-5 (i.e., 2,400 or 1,200 DH) or ii) the preselected fraction considered for introduction in the elite component (i.e., 60 or 120 DH). We did not apply a direct constraint on the proportion of selected DxE versus DExE crosses. The same DE progeny could be selected both for introduction in the elite component and for recycling in bridging.

### Preselection and Phenotyping of Donor Candidates.

For some studied bridging strategies, candidate DxE crosses were selected using donor phenotypes. In this case, diversity panel lines were phenotyped only once considering the same environmental variance as for bridging and elite progeny evaluation. Phenotypes were centered and shrunk using the observed heritability to be comparable to values predicted with a GS model. As phenotype-based evaluation of donors hinders the possibility of predicting the variance of DxE progeny, crosses were selected using a classical OCS procedure (*SI Appendix*, SI Text). We also investigated strategies with a preliminary selection of donors based on an H criterion aiming at complementing E progeny from the T-5, T-4, and T-3 generations with favorable haplotypes (“H criterion + Phenotyping” in *SI Appendix*, Table S2). H is the sum of overlapping haplotype segment values and reflects the maximum DH line performance expected after several generations of intercross and selection of the donor and elite lines (see ref. 35 and *SI Appendix*, SI Text for further details). Twenty candidate donors were selected for each generation using this criterion.

### Metrics used to Assess Scenario Performance.

At each generation, we computed the mean genetic values of all E progeny (μ) and the 10 most performing E DH lines ($\mu_{10}$) to mimic the mean value of released varieties from the elite program. Both μ and $\mu_{10}$ were reported as relative deviations to the average value observed at the end of the burn-in phase, expressed as percentage.

## Supplementary Material

Appendix 01 (PDF)

## Data Availability

Genetic data are available at https://doi.org/10.57745/58X9GT. R functions to select optimal crosses and perform simulations are available at https://forgemia.inra.fr/dimitri.sanchez1/bridging_simulation_dent_amaizing_panel.
