# Supplementary material for: Improving the use of plant genetic resources to sustain breeding programs’ efficiency
Source: Proc Natl Acad Sci U S A. 2023 Mar 27;120(14):e2205780119. doi: 10.1073/pnas.2205780119 (PMC10083577; doi:10.1073/pnas.2205780119)
Supplement: Supplementary file 1 — Appendix 01 (PDF) [file pnas.2205780119.sapp.pdf]

PNAS

Supplementary Information for

Improving the use of plant genetic resources to sustain breeding programs efficiency

Dimitri Sanchez[a], Sarah Ben Sadoun[a], Tristan Mary-Huard[a,b], Antoine Allier[c], Laurence Moreau[a], Alain Charcosset[a,*]

[a] Université Paris-Saclay, INRAE, CNRS, AgroParisTech, Génétique Quantitative et Evolution - Le Moulon, Gif-sur-Yvette, 91190, France

[b] MIA, INRAE, AgroParisTech, Université Paris-Saclay, Paris, 75005, France,

[c] Syngenta, 12 Chemin de l'Hobit, Saint-Sauveur, 31790, France

*Alain Charcosset Université Paris-Saclay, INRAE, CNRS, AgroParisTech, Génétique Quantitative et Evolution - Le Moulon, Gif-sur-Yvette, 91190, France. **Tel**: +33 01 69 33 23 35 **Email:** alain.charcosset@inrae.fr

This PDF file includes:

- Supplementary text
- Figures S1 to S6
- Tables S1 to S8
- SI References

Supplementary Information Text

H criterion computation

We consider a population of elite lines (Pop_E) and a population of potential donor lines (Pop_D). We want to select n_D^S donor lines for incorporation in Pop_E through elite x donor crosses. The haplotypic estimated breeding value matrix (HEBV) was computed as:

$$HEBV = \left(X \circ 1_N \hat{\beta}' \right) Z \; (1)$$

where X is the genotyping matrix coded in 0 or 2 of the n individuals from both populations at m markers, 1_N is an n-dimensional vector of ones, $\hat{\beta}$ is the m-dimensional vector of estimated marker effects, $\circ$ is the Hadamard pairwise product, and Z is a m x h design matrix linking the markers and the h overlapping haplotype segments. $\hat{\beta}$ was estimated by backsolving the GBLUP model calibrated on the joint TS. As in Allier et al.(1), we used haplotype segments of 100 SNPs with a 20 SNPs increment.

H was computed as:

$$\forall d \in Pop_D, \qquad H = \lambda \sum_{j=1}^{h} \max\left(HEBV_{d,j}, \max_{i \in Pop_E} \left(HEBV_{i,j}\right) \right) (2)$$

where λ= number of SNPs per segment/number of SNPs increment. λ is a scaling parameter taking into account the overlap between segments. The donor line with the highest H value is added to the Pop_E set. The procedure is repeated until reaching n_D^S donor lines.

Optimal Cross Selection

OCS aims at selecting a cross list that maximizes the expected global performance in the progeny (V) under a genomewide genetic diversity constraint (D). We used it to select the elite crosses (ExE for strategy without introductions or ExE and DExE for strategies with introductions). The number of crosses (nc_E) was predetermined and set to 20, 15 or 10 according to breeding resource allocations. At each generation, we aimed at solving the following constrained optimization problem:

$$\max V_{nc_E}$$
$$with \; D_{nc_E} > He(t), (1)$$

where $He(t)$ is the diversity constraint at generation t ($t \in [0, t^*]$, t^* was set to 60). $He(t)$ determines the targeted elite diversity trajectory. As in (2), we considered a linear trajectory:

$$He(t) = He^0 + \frac{t}{t^*}(He^* - He^0) \ (2)$$

where He^0 is the initial neutral diversity at the end of the burn-in and $He^*$is the final diversity targeted at t^*. We considered $He^* = 0.01$ to define the linear decrease trajectory and $He^* = He^0$ to maintain a constant diversity (see SI Appendix, Table S2). He^0 is defined as :

$$He^0 = \frac{1}{m}\sum_{j=1}^{m} 2p_j^0(1 - p_j^0) \ (3)$$

where m is the neutral marker number and p_j^0 is the frequency of the referent allele at the end of burn-in.

To compute V_{nc_E} and D_{nc_E} values, we followed the two OCS approaches presented by Woolliams et al. (3) and Allier et al (5), respectively. These differ in the estimations of the contribution of each parent genome to the progeny (c) and the value of crosses. The first one, called "classical OCS" above, is commonly used in animal breeding (3). In this case, the performance of a cross is defined as the mean parental performance (GEBVs or phenotype) and V_{nc_E} is the mean performance of the nc_E crosses. D_{nc_E} is estimated using parental contribution defined as:

$$c = \frac{1}{nc_E}(Z_1c_1 + Z_2c_2) \ (4)$$

where Z_1 (respectively Z_2) is a design matrix linking the N potential parents to the first (respectively second) parent in the cross list, c_1 (respectively c_2) is a nc_E*1 dimensional vector containing the contribution of the first (respectively second) parent to the progeny of each cross. As only pedigree information is considered, all values in c_1 (respectively c_2) are set to 0.5.

With this approach, the optimization problem (4) becomes:

$$\max\left\{\frac{1}{nc_E}\sum_{j\in[1,nc_E]}\frac{P_{1j} + P_{2j}}{2}\right\}$$

$$1 - c'K_Nc > He(t) \ (5)$$

where P_{1j} (respectively P_{2j}) is the performance of the first (respectively second) parent of cross j and K_N is an NxN IBS matrix compute as:

$$K_N = \frac{1}{2}\left(\frac{1}{m}X_NX_N' + 1\right) \ (6)$$

where X_N is the Nxm genotyping matrix of candidate parents coded in -1 or 1. The relationship between the expected diversity in progeny and the IBS parental matrix is detailed in Allier et al. (2)

The second OCS approach, based on UCPC, considers (i) expected variance generated by the cross and (ii) expected consequences of within family selection in the progeny on parental contribution. c_1 and c_2 in (6) vary here according to the expected parental genome after selection in the progeny (see (2)). The performance of a cross is then given by its UC value instead of the mean parental performance. The optimization problem (4) is then described by:

$$\max\left\{\frac{1}{nc_E}\sum_{j\in[1,nc_E]} UC_j\right\}$$

$$1- \ c'K_N c > He(t) \ (7)$$

In case of donor introductions, we chose the nc_B bridging crosses conditionally to the nc_E crosses selected for elite. We selected the nc_B bridging crosses which maximize the following score computed on the full set of nc crosses (here $nc = \ nc_E + nc_B = 20$):

$$Score_{VD} = \alpha V^* + (1-\alpha) D^* \ (8)$$

where $V^* = \frac{V_{nc}-V_{nc_D}}{V_{nc_V}-V_{nc_D}}$ and $D^* = \frac{D_{nc}-D_{nc_V}}{D_{nc_D}-D_{nc_V}}$ with nc_V and nc_D the lists of crosses (nc_E fixed elite crosses and nc_B variable bridging crosses), that maximize the performance (V) or the diversity (D), respectively. V_{nc} and D_{nc} were computed as described above for the UCPC based OCS approach, except when selecting candidate donor based on phenotyping. $\alpha \in [0,1]$ is the relative weight given to performance compared to diversity (here $\alpha = 0.3$). We used forward and swapping algorithms to find a solution to (1) and (8) presented in the next section.

Algorithms implemented to perform optimal cross selection

We detail here the implementation of an algorithm that solves the constrained optimization problem posed by the OCS described in Wooliams and al (3). The objective is to select a cross list that maximizes the expected global performance in the progeny (V) under a genomewide genetic diversity constraint (D).

Definition of the problem to optimize We consider N potential parents. For all crosses ($\frac{N(N-1)}{2}$), the contribution to each parent was determined and a performance value was attributed (using parental mean value or a usefulness criterion). We want to determine the list of nc crosses that solve the following constrained optimization problem:

$$\max V_{nc} = \frac{1}{nc} \sum_{j \in [1,nc]} v_j$$

$$with\ D_{nc} = 1 - c' K_N c > He_{min}\ (1)$$

where v_j is the performance value of the cross j, K_N is an NxN Identity by State (IBS)matrix, He_{min} is the minimal genetic diversity required in the progeny, c is the N-dimensional vector of parental contribution to the progeny:

$$c = \frac{1}{nc}(Z_1 c_1 + Z_2 c_2)\ (2)$$

where Z_1 (respectively Z_2) is a design matrix linking the N potential parents to the first (respectively second) parent in the cross list, c_1 (respectively c_2) is a nc dimensional vector containing the contribution of the first (respectively second) parent to progeny.

Evolution of D when a cross is added to an initial cross list We consider two lists of crosses $A = (cross_1, cross_2, \dots, cross_n)$ $B = (cross_1, cross_2, \dots, cross_n, cross_{n+1})$ $(A \subset B)$. We have:

$$c_B = \frac{n}{n+1}\left[c_A + \frac{1}{n}\left(Z^B_{1\,(.,n+1)} c^B_{1(n+1)} + Z^B_{2\,(.,n+1)} c^B_{2(n+1)}\right)\right]\ (3)$$

where $Z^B_{1\,(.,n+1)}$ (respectively $Z^B_{2\,(.,n+1)}$) is the last column of the matrix Z_1 (respectively Z_2) and $c^B_{1(n+1)}$ (respectively $c^B_{2(n+1)}$) is the $n+1^{th}$ element of the vector c_1 (respectively c_2) in (2) for the list B.

We notice that:

$$D_B = 1 - \left[\left(\frac{n}{n+1}\right)^2 (1 - D_A) + \left(\frac{1}{n}\right)^2 Y' K_N Y + \frac{2n}{(n+1)^2}(1 - D_A) K_N Y\right]\ (4)$$

where $Y = Z^B_{1\,(.,n+1)} c^B_{1(n+1)} + Z^B_{2\,(.,n+1)} c^B_{2(n+1)}$

Consequently, D can be updated efficiently when adding a new cross to the list.

Step 1: Definition of a cross list using a forward approach To initialize the procedure, we select a random list of 5 crosses among candidate parents. Using (4), we determine all the crosses leading to $D > He_{min}$

when they are added individually to the initial list. If several crosses answer this condition, we add to the list the cross with the highest value. We continue this process until reaching a list of nc crosses. If no cross allows respecting the condition $D > He_{min}$ at a given step before reaching nc, we start again the procedure with another random initial list. We authorize up to 10 000 initializations for a given initial length of cross list (set at 5 at the first step). If no initialization with this length leads to a complete list of nc crosses filling the condition, we consider initializations with one more cross. We continue the procedure until finding a cross-list filling the condition or reaching an initial number of crosses higher than nc. In this latter case, we consider that the algorithm failed.

Step 2: Improving the performance of the selected cross list using a swapping approach. To minimize the impact of the random initial list, we implemented a swapping algorithm to explore a larger number of cross lists. From the cross list determined by step 1, we remove the cross with the lowest value and apply the forward algorithm described in step 1 to find the best possible substitute. If the value of this cross is superior to the initial one, the replacement is accepted. The procedure is repeated until no cross is found with a higher value than the removed cross. After that, we try to replace the cross with the second lowest value and so on. We consider that we found a solution to the optimization problem when no further substitution led to an improvement of V.

Adaptation to the $\boldsymbol{Score_{VD}}$ computation We consider a population of elite lines (E individuals) and a population of potential donor lines (D individuals). After having selected nc_E crosses to generate the E progeny and we want to here to complete the list with nc_B DxE introductions crosses to create a full list of nc crosses. We use the following score:

$$Score_{VD} = \alpha V^* + (1-\alpha)D^* \ (5)$$

where $V^* = \frac{V_{nc}-V_{nc_D}}{V_{nc_V}-V_{nc_D}}$ and $D^* = \frac{D_{nc}-D_{nc_V}}{D_{nc_D}-D_{nc_V}}$ with nc_V and nc_D the lists of crosses, including the nc_E selected elite crosses and nc_B bridging crosses, that maximize the performance (V) or the diversity (D). $\alpha \in [0,1]$ is the relative weight given to performance compared to diversity.

nc_V is easily determined by adding the nc_B crosses with the highest values to the nc_E crosses. nc_D is obtained by starting from the nc_E crosses, and adding iteratively the DxE cross which maximizes D, until reaching nc .

To determine the full list, we randomly select nc_D DxE crosses to complete the nc_E crosses. We then remove each DxE cross and replace it which the cross which maximizes $Score_{VD}$. Among the nc_D obtained lists, we keep the one which lead to the highest $Score_{VD}$ value. The procedure is continued until the stabilization of the score.

The same approach was extended to consider the recycling of bridging progeny (DE individuals). In this case, the nc_B crosses are selected among DxE and DExE crosses.

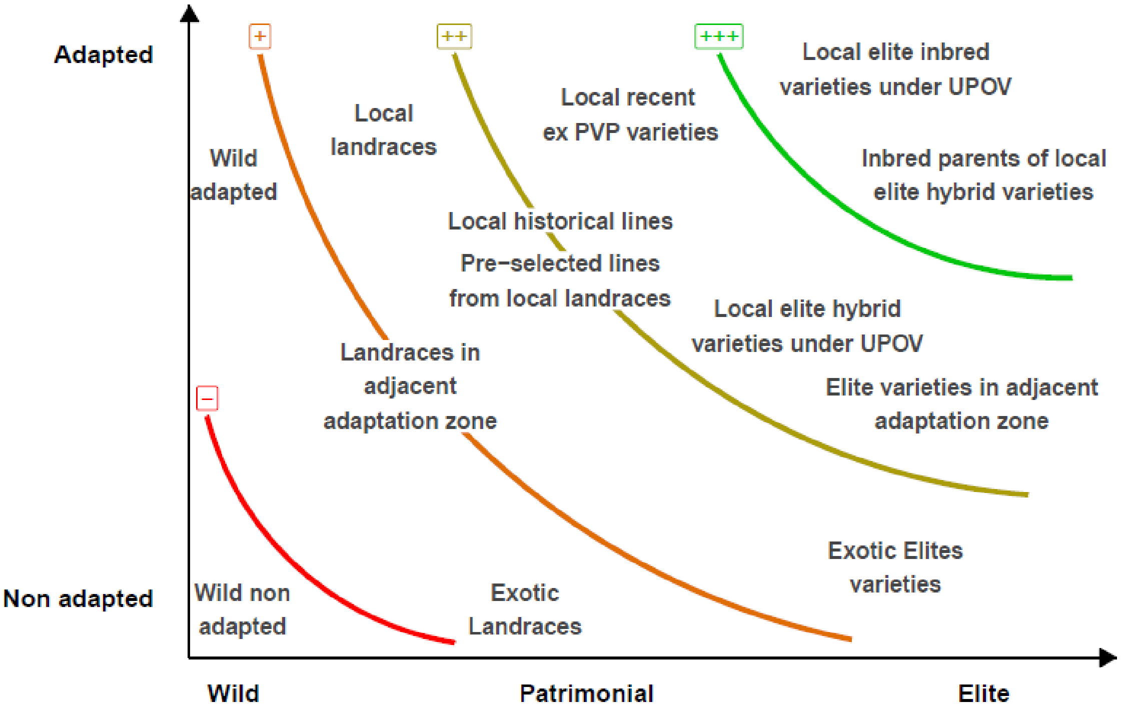

Fig. S1 Typology of genetic resources available as diversity donors. Level curves indicate readiness for use in breeding programs (from – to +++). UPOV: Union for the Protection of New Varieties of Plants, PVP: Plant Variety Protection

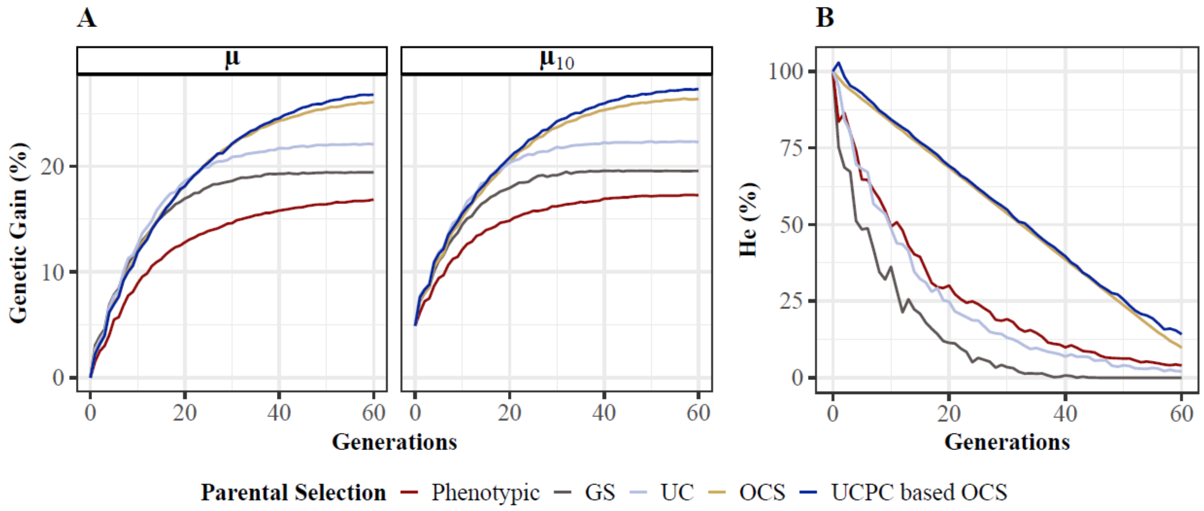

Fig. S2 Evaluation of parental selection strategies in a closed breeding program A) Mean genetic gain for elite population ($\boldsymbol{\mu}$) and for the 10 best individuals ($\boldsymbol{\mu_{10}}$) from the end of the burn-in phase. B) Elite neutral Nei diversity (He) in percentage of the neutral Nei diversity at the end of the burn-in phase. Colours indicate the different parental selection options. GS: genomic selection, UC: usefulness criterion, OCS: Optimal Cross selection, UCPC: uselfulness criterion parental contribution

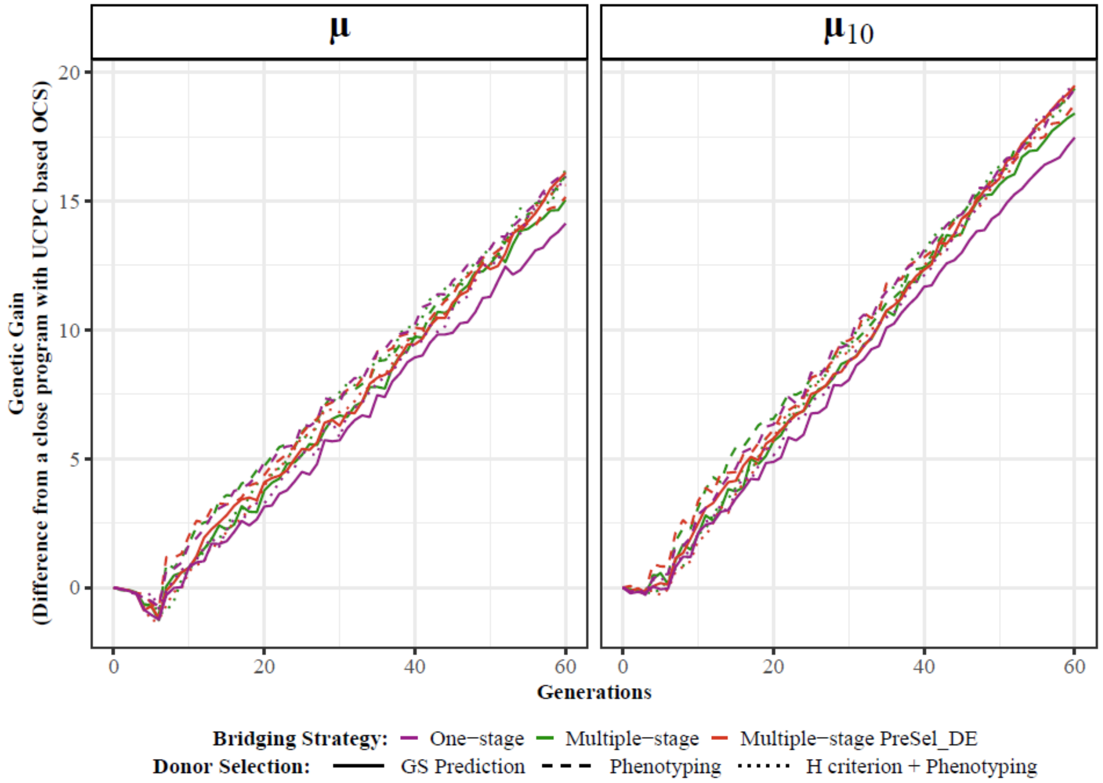

Fig. S3. Comparison of one-stage and multiple-stage bridging strategies with different donor selection options. μ: mean genetic gain for elite population. μ_{10}: mean genetic value for the 10 best individuals. Values are reported as deviations from a closed program managed with UCPC based OCS. "One-stage", "Multiple-stage" and "Multiple-stage PreSel_DE": bridging strategies. "GS Prediction", "Phenotyping" and "H criterion + Phenotyping": donor selection options. (Resource allocation: $\mathbf{15}\boldsymbol{C_E} + \mathbf{5}\boldsymbol{C_B}$)

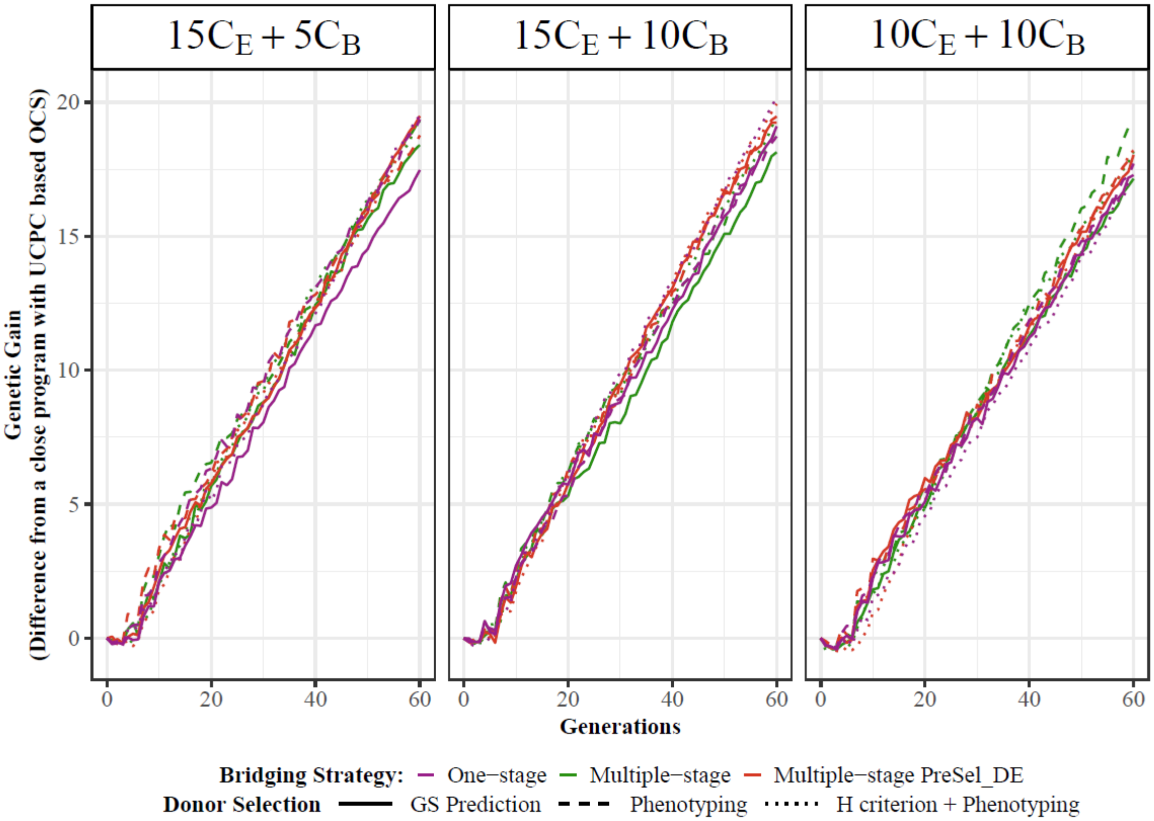

Fig. S4. Comparison of one-stage and multiple-stage bridging strategies with different donor selection options and resource allocations. Genetic gain refers to the mean genetic value for the 10 best individuals (μ_{10}). It is reported as deviations from a closed program managed with UCPC based OCS. "$15C_E + 5C_B$", "$15C_E + 10C_B$" and "$10C_E + 10C_B$" indicate resource allocation, expressed as the number of crosses devoted to the bridging and elite populations, respectively "One-stage", "Multiple-stage" and "Multiple-stage PreSel_DE": bridging strategies. "GS Prediction", "Phenotyping" and "H criterion + Phenotyping": donor selection options.

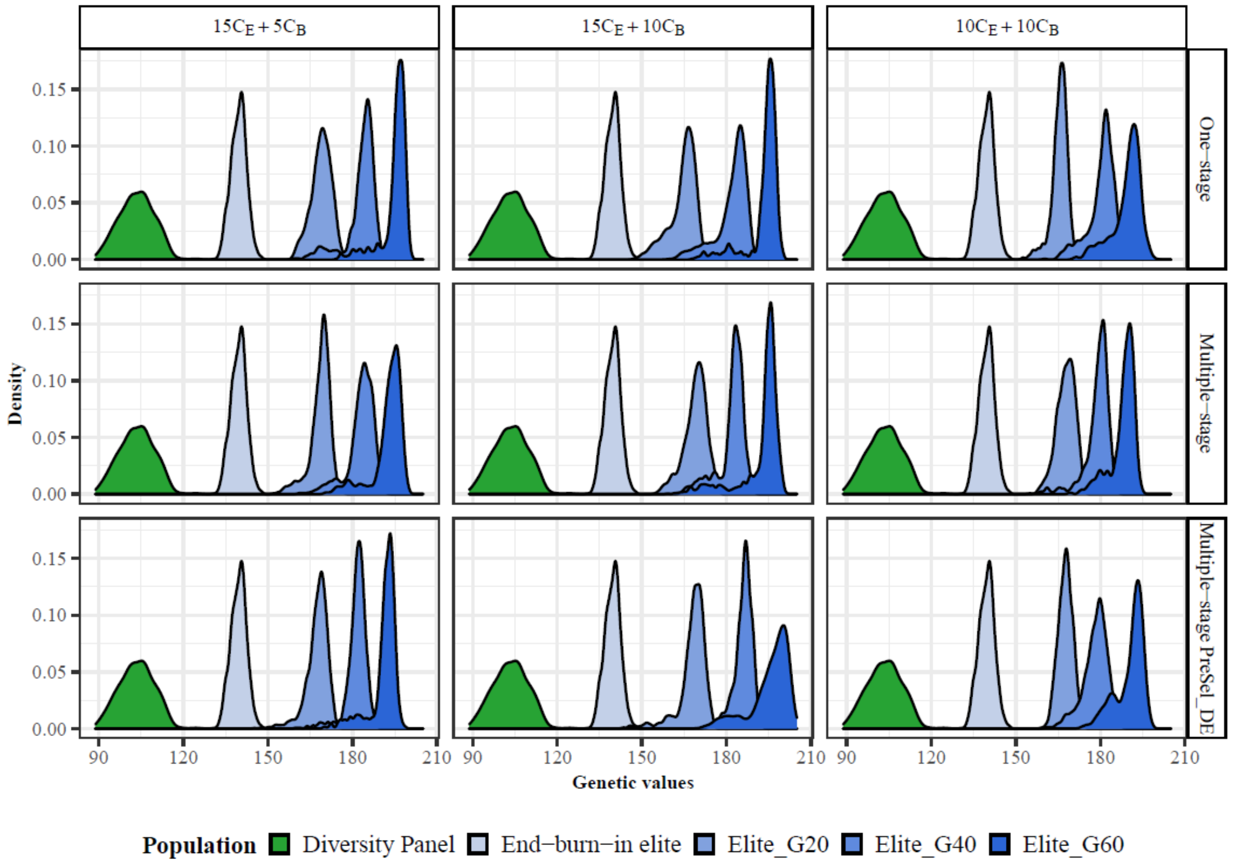

Fig. S5 Distributions of genetic values in the diversity panel and different generations of the elite component. Distributions are shown for a random replicate. The diversity panel and end-burn-in-elite are common between strategies. **"**$15C_E + 5C_B$**", "**$15C_E + 10C_B$" and "$10C_E + 10C_B$": resource allocation. ."One-stage", "Multiple-stage" and "Loop NoPreSel_DE": bridging strategies

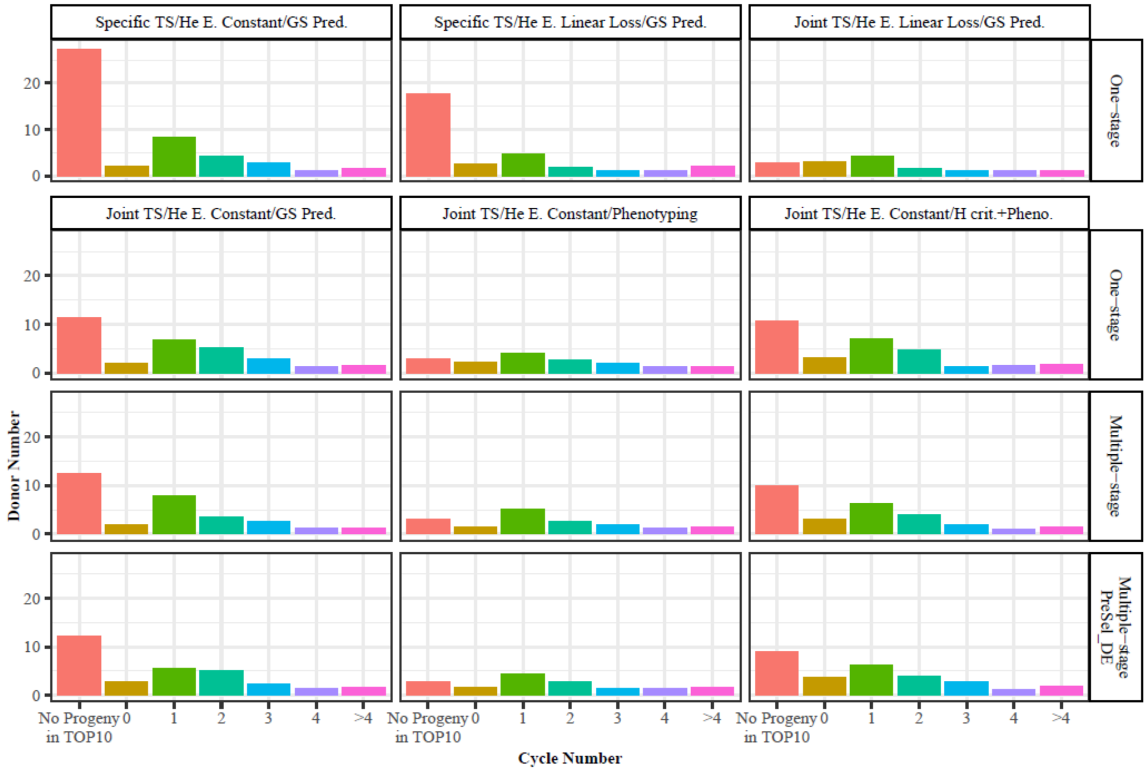

Fig. S6 Classification of donors introduced in elite component according the number of additional crosses before their progeny reaches the top 10 lines. The numbers of donors are established at generation 60 and averaged over the 10 replicates

Table S1. Performance for different cross selection methods in closed breeding programs at generation 15 and 60

		Parental Selection					
**Generation**	**Mean**	**Phenotypic**	**GS**	**UC**	**OCS**	**UCPC based OCS**	**UCPC based OCS (He constant)**
**15**	$\boldsymbol{\mu}$	**11.21** (0.64)	**15.19** (0.83)	**16.40** (0.80)	**15.51** (0.70)	**15.28** (0.74)	**13.91** (0.71)
	$\boldsymbol{\mu_{10}}$	**13.97** (0.63)	**16.71** (0.87)	**18.54** (0.91)	**18.15** (0.79)	**18.45** (0.81)	**18.26** (0.81)
**60**	$\boldsymbol{\mu}$	**16.86** (0.87)	**19.43** (1.14)	**22.09** (1.23)	**26.08** (1.18)	**26.79** (1.57)	-
	$\boldsymbol{\mu_{10}}$	**17.26** (0.95)	**19.56** (1.14)	**22.30** (1.25)	**26.38** (1.21)	**27.32** (1.60)	-

$\boldsymbol{\mu}$: Mean genetic gain for elite population from the end of the burn-in phase. $\boldsymbol{\mu_{10}}$: Mean genetic gain for the 10 best individuals from the end of the burn-in phase. GS: genomic selection, UC: usefulness criterion, OCS: Optimal Cross selection, UCPC: usefulness criterion parental contribution. Values between parentheses indicate standard errors of the estimated values.

Table S2. Overview of simulated bridging breeding programs

Bridging strategy	GS model calibration	Elite diversity management	Donor selection guideline	breeding resource allocation		
				$15C_E + 5C_B$	$15C_E + 10C_B$	$10C_E + 10C_B$
One-stage	Specific TS	He constant	GS Prediction	✓	□	□
		He Linear Loss		✓	□	□
	Joint TS	He constant	GS Prediction	✓	✓	✓
		He Linear Loss		✓	□	□
		He constant	Phenotyping	✓	✓	✓
			H criterion + Phenotyping	✓	✓	✓
Multiple-stage	Joint TS	He constant	GS Prediction	✓	✓	✓
		He Linear Loss		✓	□	□
		He constant	Phenotyping	✓	✓	✓
			H criterion + Phenotyping	✓	✓	✓
Multiple-stage with DE progenies pre-selection	Joint TS	He constant	GS Prediction	✓	✓	✓
		He Linear Loss		✓	□	□
		He constant	Phenotyping	✓	✓	✓
			H criterion + Phenotyping	✓	✓	✓

Each row corresponds to a simulated strategy. Resource allocation indicates the number of crosses devoted to bridging and elite components, respectively. Elite crosses are represented in all cases by 80 progenies, bridging crosses are represented by 80 progenies for strategies $15C_E + 5C_B$ and $10C_E + 10C_B$vs. 40 for $15C_E + 10C_B$. Marks indicate the combination of strategies and resource allocations which were evaluated. Values between parentheses indicate standard errors of the estimated values.

Table S3. Performance for closed programs and one-stage bridging programs (resource allocation: "5/15") at generation 15 and 60

Generation	Strategy	Elite diversity management	$\boldsymbol{\mu}$	$\boldsymbol{\mu_{10}}$
15	No Intro GS		**15.19** (0.83)	**16.71** (0.87)
	No Intro UCPC based OCS	He Linear Loss	**15.28** (0.74)	**18.45** (0.81)
	One-Stage Bridging Specific TS	He constant	**15.67** (0.84)	**20.15** (0.88)
		He Linear Loss	**16.21** (0.92)	**20.27** (1.02)
	One-Stage Bridging Joint TS	He constant	**17.07** (1.11)	**21.90** (1.18)
		He Linear Loss	**17.78** (0.83)	**21.92** (0.92)
60	No Intro GS		**19.43** (1.14)	**19.56** (1.14)
	No Intro UCPC based OCS	He Linear Loss	**26.79** (1.57)	**27.32** (1.60)
	One-Stage Bridging Specific TS	He constant	**38.42** (1.60)	**43.90** (1.69)
		He Linear Loss	**34.40** (1.65)	**37.45** (1.47)
	One-Stage Bridging Joint TS	He constant	**40.92** (1.30)	**44.79** (1.40)
		He Linear Loss	**39.46** (1.67)	**41.70** (1.50)

$\boldsymbol{\mu}$: Mean genetic gain for elite population from the end of the burn-in phase. $\boldsymbol{\mu_{10}}$: Mean genetic gain for the 10 best individuals from the end of the burn-in phase. GS: genomic selection, OCS: Optimal Cross selection, TS: Training Set. "No intro" indicates a closed breeding program. Values between parentheses indicate standard errors of the estimated values.

Table S4. Performance for one-stage and multiple-stage bridging programs (resource allocation: "5/15") at generations 15 and 60.

Generation	Strategy	Elite diversity management	$\boldsymbol{\mu}$	$\boldsymbol{\mu_{10}}$
15	One-stage	He constant	**17.07** (1.11)	**21.90** (1.18)
	Multiple-stage	He constant	**17.52** (1.08)	**22.18** (1.16)
		He Linear Loss	**18.44** (1.07)	**22.64** (1.10)
	Multiple-stage PreSel_DE	He constant	**18.09** (1.15)	**22.59** (1.20)
		He Linear Loss	**18.79** (0.90)	**22.75** (0.98)
60	One-stage	He constant	**40.92** (1.30)	**44.79** (1.40)
	Multiple-stage	He constant	**41.84** (1.32)	**45.72** (1.38)
		He Linear Loss	**38.19** (1.32)	**40.04** (1.44)
	Multiple-stage PreSel_DE	He constant	**42.89** (1.49)	**46.80** (1.54)
		He Linear Loss	**38.79** (1.28)	**41.04** (1.41)

$\boldsymbol{\mu}$: Mean genetic gain for elite population from the end of the burn-in phase. $\boldsymbol{\mu_{10}}$: Mean genetic gain for the 10 best individuals from the end of the burn-in phase. Values between parentheses indicate standard errors of the estimated values. Donors are pre-selected based on their genetic values predicted with the Joint TS genomic selection model ("GS prediction").

Table S5. Performance for one-stage and multiple-stage bridging programs with different resource allocations and donor selection options at generations 15 and 60.

			Donor Selection					
**Generation**	**Strategy**	**Allocation**	**GS Prediction**		**Phenotyping**		**H criterion + Phenotyping**	
			$\boldsymbol{\mu}$	$\boldsymbol{\mu_{10}}$	$\boldsymbol{\mu}$	$\boldsymbol{\mu_{10}}$	$\boldsymbol{\mu}$	$\boldsymbol{\mu_{10}}$
15	One-stage	$15C_E + 5C_B$	**17.07** (1.11)	**21.90** (1.18)	**18.51** (1.05)	**23.07** (1.15)	**17.52** (0.91)	**22.25** (1.00)
		$15C_E + 10C_B$	**18.25** (1.04)	**22.95** (1.09)	**17.95** (1.01)	**22.48** (1.02)	**17.88** (0.75)	**22.62** (0.83)
		$10C_E + 10C_B$	**17.82** (0.97)	**22.25** (1.01)	**18.03** (0.89)	**22.65** (0.87)	**16.96** (0.85)	**21.20** (0.85)
	Multiple-stage	$15C_E + 5C_B$	**17.52** (1.08)	**22.18** (1.16)	**18.86** (0.99)	**23.89** (1.07)	**17.66** (0.98)	**22.32** (1.12)
		$15C_E + 10C_B$	**17.94** (1.02)	**22.30** (1.09)	**18.31** (1.01)	**22.77** (1.07)	**17.94** (0.81)	**22.83** (0.95)
		$10C_E + 10C_B$	**17.30** (1.11)	**22.13** (1.14)	**17.64** (1.17)	**22.38** (1.31)	**17.10** (0.77)	**21.85** (0.79)
	Multiple-stage PreSel_DE	$15C_E + 5C_B$	**18.09** (1.15)	**22.59** (1.20)	**18.50** (1.06)	**22.94** (1.25)	**17.48** (0.74)	**22.09** (0.90)
		$15C_E + 10C_B$	**17.87** (1.02)	**22.35** (0.98)	**17.95** (1.07)	**22.49** (1.22)	**17.24** (0.89)	**22.03** (0.99)
		$10C_E + 10C_B$	**17.66** (1.11)	**22.77** (1.23)	**17.92** (1.01)	**22.31** (1.08)	**16.65** (0.61)	**21.22** (0.78)
60	One-stage	$15C_E + 5C_B$	**40.92** (1.30)	**44.79** (1.40)	**42.69** (1.60)	**46.67** (1.59)	**42.73** (1.53)	**46.78** (1.56)
		$15C_E + 10C_B$	**41.74** (1.49)	**46.42** (1.40)	**42.24** (1.61)	**46.04** (1.59)	**42.88** (1.60)	**47.50** (1.58)
		$10C_E + 10C_B$	**40.27** (1.87)	**44.60** (1.87)	**40.52** (1.80)	**45.03** (1.80)	**40.32** (1.31)	**44.45** (1.30)
	Multiple-stage	$15C_E + 5C_B$	**41.84** (1.32)	**45.72** (1.38)	**42.77** (1.78)	**46.69** (1.70)	**43.02** (1.53)	**46.61** (1.55)
		$15C_E + 10C_B$	**41.07** (1.44)	**45.45** (1.45)	**41.88** (1.64)	**46.41** (1.62)	**42.55** (1.56)	**46.73** (1.49)
		$10C_E + 10C_B$	**40.37** (1.70)	**44.46** (1.71)	**42.32** (2.09)	**46.15** (2.18)	**41.59** (1.37)	**45.47** (1.37)
	Multiple-stage PreSel_DE	$15C_E + 5C_B$	**42.89** (1.49)	**46.80** (1.54)	**41.95** (1.73)	**46.08** (1.91)	**42.41** (1.39)	**46.67** (1.31)
		$15C_E + 10C_B$	**42.05** (1.55)	**46.55** (1.46)	**42.35** (1.59)	**46.78** (1.71)	**42.61** (1.71)	**47.25** (1.54)
		$10C_E + 10C_B$	**41.02** (1.39)	**45.35** (1.41)	**40.82** (1.83)	**45.38** (1.96)	**41.29** (1.63)	**45.53** (1.62)

$\boldsymbol{\mu}$: Mean genetic gain for elite population from the end of the burn-in phase. $\boldsymbol{\mu_{10}}$: Mean genetic gain for the 10 best individuals from the end of the burn-in phase. For the displayed strategies, the diversity is maintained constant in the elite component. Values between parentheses indicate standard errors of the estimated values.

Table S6. Percentages of QTLs fixed for the negative or favourable allele and not fixed QTLs at generation 60 in closed breeding programs

	QTL Percent			Weighted QTL Percent		
**Parental Selection**	**Favourable Allele**	**Negative Allele**	**Not fixed**	**Favourable Allele**	**Negative Allele**	**Not fixed**
Phenotypic	**59.78** (0.59)	**39.20** (0.64)	**1.02** (0.15)	**65.30** (0.74)	**33.95** (0.79)	**0.75** (0.15)
GS	**61.18** (0.46)	**38.46** (0.47)	**0.36** (0.05)	**66.58** (0.73)	**33.17** (0.73)	**0.26** (0.05)
UC	**61.80** (0.43)	**37.66** (0.44)	**0.54** (0.11)	**67.53** (0.68)	**32.14** (0.67)	**0.32** (0.08)
OCS	**62.65** (0.46)	**35.82** (0.57)	**1.53** (0.22)	**68.83** (0.70)	**30.44** (0.77)	**0.73** (0.15)
UCPC based OCS	**62.58** (0.45)	**35.28** (0.64)	**2.14** (0.22)	**68.81** (0.75)	**30.01** (0.90)	**1.18** (0.19)

QTLs are classified into three groups: fixed for the negative allele, fixed for the favourable allele and not fixed. The repartition across the groups are given by "QTL percent". These values are weighted using QTL effects: "Weighted QTL percent" are the sums by groups of absolute QTL effects divided by the total sum of absolute QTL effects. GS: genomic selection, UC: usefulness criterion, OCS: Optimal Cross selection. Values between parentheses indicate standard errors of the estimated values.

Table S7. Percentages of QTLs fixed for the negative or favourable allele and not fixed QTLs at generation 60 in open breeding programs

Allocation	Strategy	GS Model	He Management	Donor Selection Strategy	QTL Percent: Favourable Allele	QTL Percent: Negative Allele	QTL Percent: Not fixed	Weighted QTL percent: Favourable Allele	Weighted QTL percent: Negative Allele	Weighted QTL percent: Not fixed
$15C_E + 5C_B$	One-stage	Specific TS	He constant	GS Prediction	**42.64** (1.61)	**19.98** (0.95)	**37.38** (2.50)	**49.51** (1.87)	**15.77** (0.92)	**34.72** (2.66)
			He Linear Loss		**55.91** (1.49)	**29.05** (0.90)	**15.04** (2.30)	**63.10** (1.67)	**23.02** (0.90)	**13.88** (2.41)
		Joint TS	He constant	GS Prediction	**47.85** (1.00)	**21.53** (0.69)	**30.62** (1.52)	**56.46** (1.01)	**16.54** (0.64)	**27.00** (1.48)
			He Linear Loss		**61.49** (1.10)	**29.49** (0.67)	**9.02** (1.67)	**69.89** (1.34)	**22.85** (0.48)	**7.26** (1.71)
			He constant	Phenotyping	**46.75** (1.59)	**19.68** (0.67)	**33.57** (2.20)	**55.26** (1.72)	**15.24** (0.51)	**29.50** (2.11)
				H crit. + Phenotyping	**47.21** (1.31)	**20.31** (0.61)	**32.48** (1.80)	**55.78** (1.31)	**15.43** (0.52)	**28.79** (1.69)
	Multiple-stage	Joint TS	He constant	GS Prediction	**46.57** (0.86)	**19.85** (0.55)	**33.58** (1.27)	**55.12** (0.89)	**15.34** (0.51)	**29.54** (1.10)
			He Linear Loss		**59.11** (1.58)	**29.38** (0.56)	**11.51** (2.00)	**67.43** (1.83)	**23.12** (0.41)	**9.45** (2.01)
			He constant	Phenotyping	**47.50** (1.31)	**20.41** (0.85)	**32.09** (2.14)	**56.09** (1.38)	**15.99** (0.77)	**27.92** (2.07)
				H crit. + Phenotyping	**49.24** (1.37)	**20.90** (1.06)	**29.86** (2.20)	**58.72** (1.50)	**15.98** (0.87)	**25.30** (2.09)
	Multiple-stage NoPreSel_DE	Joint TS	He constant	GS Prediction	**47.46** (1.33)	**19.86** (0.82)	**32.68** (1.97)	**56.44** (1.40)	**15.23** (0.82)	**28.33** (1.88)
			He Linear Loss		**60.82** (1.39)	**29.05** (0.76)	**10.13** (1.97)	**69.06** (1.63)	**22.83** (0.60)	**8.11** (1.97)
			He constant	Phenotyping	**45.71** (1.93)	**19.49** (1.00)	**34.80** (2.87)	**54.29** (2.25)	**15.45** (0.90)	**30.27** (3.05)
				H crit. + Phenotyping	**46.27** (1.53)	**20.08** (0.79)	**33.65** (2.23)	**54.18** (1.42)	**15.33** (0.72)	**30.48** (2.01)
$15C_E + 10C_B$	One-stage	Joint TS	He constant	GS Prediction	**46.17** (2.09)	**20.08** (1.14)	**33.75** (3.15)	**54.92** (2.29)	**15.24** (0.88)	**29.84** (2.94)
				Phenotyping	**46.74** (1.87)	**19.92** (0.97)	**33.34** (2.77)	**54.73** (2.15)	**15.79** (0.83)	**29.47** (2.85)
				H crit. + Phenotyping	**44.80** (1.92)	**19.59** (0.91)	**35.61** (2.73)	**53.05** (2.14)	**15.03** (0.70)	**31.92** (2.68)
	Multiple-stage	Joint TS	He constant	GS Prediction	**47.91** (1.43)	**20.67** (0.89)	**31.42** (2.21)	**56.45** (1.58)	**16.01** (0.72)	**27.53** (2.11)
				Phenotyping	**45.91** (1.98)	**19.83** (0.90)	**34.26** (2.81)	**54.29** (2.33)	**15.48** (0.81)	**30.23** (2.99)
				H crit. + Phenotyping	**45.79** (1.30)	**19.38** (0.89)	**34.83** (2.13)	**53.89** (1.51)	**14.90** (0.76)	**31.21** (2.15)
	Multiple-stage NoPreSel_DE	Joint TS	He constant	GS Prediction	**45.37** (1.32)	**20.23** (0.66)	**34.40** (1.91)	**53.30** (1.47)	**15.23** (0.68)	**31.47** (2.11)
				Phenotyping	**44.62** (1.24)	**19.42** (0.81)	**35.96** (1.93)	**52.83** (1.31)	**14.99** (0.70)	**32.18** (1.88)
				H crit. + Phenotyping	**46.84** (1.62)	**20.26** (0.89)	**32.90** (2.45)	**55.45** (1.83)	**15.27** (0.69)	**29.29** (2.45)
$10C_E + 10C_B$	One-stage	Joint TS	He constant	GS Prediction	**48.03** (1.67)	**21.17** (0.74)	**30.80** (2.38)	**56.49** (1.70)	**16.33** (0.51)	**27.17** (2.12)
				Phenotyping	**45.25** (1.16)	**20.27** (0.79)	**34.48** (1.90)	**53.33** (1.33)	**15.62** (0.70)	**31.05** (1.95)
				H crit. + Phenotyping	**47.06** (1.24)	**20.27** (0.86)	**32.67** (1.99)	**54.95** (1.36)	**15.54** (0.76)	**29.52** (1.98)
	Multiple-stage	Joint TS	He constant	GS Prediction	**46.91** (1.22)	**20.52** (0.85)	**32.57** (1.98)	**54.99** (1.33)	**15.49** (0.76)	**29.52** (1.87)
				Phenotyping	**48.69** (2.03)	**20.98** (0.96)	**30.33** (2.85)	**57.46** (2.19)	**15.77** (0.67)	**26.77** (2.60)
				H crit. + Phenotyping	**48.47** (1.14)	**21.01** (0.93)	**30.52** (1.98)	**58.01** (1.13)	**15.68** (0.73)	**26.31** (1.75)
	Multiple-stage NoPreSel_DE	Joint TS	He constant	GS Prediction	**46.83** (1.73)	**20.03** (0.61)	**33.14** (2.15)	**55.18** (1.94)	**15.20** (0.61)	**29.62** (2.28)
				Phenotyping	**45.97** (1.21)	**19.70** (0.95)	**34.33** (2.10)	**54.26** (1.27)	**15.42** (0.71)	**30.32** (1.90)
				H crit. + Phenotyping	**49.21** (1.23)	**21.09** (0.79)	**29.70** (1.96)	**57.54** (1.27)	**16.06** (0.69)	**26.39** (1.84)

QTLs are classified into three groups: fixed for the negative allele, fixed for the favourable allele and not fixed. The repartition between the groups is indicated by “QTL percent”. “Weighted QTL percent” indicates values weighted by QTL effects: sums by groups of absolute QLT effects are divided by the total sum of absolute QTL effects. Values between parentheses indicate standard errors of the estimated values.

Table S8 Contribution of the selected donor progenies to the breeding components

					Generation 15							Generation 60						
Allocation	Strategy	GS Model	He Management	Donor Selection Strategy	N_{SD}	N_{ID}	N_{CD}	$N_{CDtop10}$	$\frac{CD}{ID}$ (%)	$\frac{CDtop10}{ID}$ (%)	% Elite Genome	N_{SD}	N_{ID}	N_{CD}	$N_{CDtop10}$	$\frac{CD}{ID}$ (%)	$\frac{CDtop10}{ID}$ (%)	% Elite Genome
$15C_E + 5C_B$	One-stage	Specific TS	He constant	GS Prediction	**35.2** (1.7)	**6.3** (0.5)	**3.8** (0.2)	**2.5** (0.2)	**63.2** (5.9)	**40.9** (3.1)	**15.5** (1.8)	**114.7** (2.5)	**45.6** (1.7)	**18.8** (1.5)	**16.1** (1.2)	**41.1** (2.7)	**35.1** (2.0)	**45.7** (1.0)
			He Linear Loss		**33.3** (1.9)	**5.9** (0.8)	**2.9** (0.3)	**1.5** (0.5)	**55.4** (7.2)	**35.0** (10.6)	**11.4** (1.9)	**111.5** (1.6)	**27.7** (1.5)	**8.5** (0.8)	**7.5** (0.7)	**32.0** (3.9)	**28.3** (3.7)	**37.4** (2.6)
		Joint TS	He constant	GS Prediction	**36.3** (1.0)	**4.1** (0.5)	**3.4** (0.4)	**2.2** (0.3)	**86.5** (4.7)	**63.2** (10.3)	**19.1** (1.9)	**119.0** (1.8)	**28.4** (1.7)	**18.4** (1.4)	**15.6** (1.3)	**64.6** (2.8)	**54.6** (2.5)	**46.6** (1.2)
			He Linear Loss		**36.8** (1.6)	**3.9** (0.3)	**3.0** (0.2)	**2.4** (0.3)	**80.2** (5.9)	**65.2** (9.1)	**19.0** (2.7)	**111.5** (2.4)	**11.0** (0.9)	**7.9** (0.5)	**7.7** (0.5)	**73.8** (4.5)	**72.2** (5.2)	**39.7** (3.5)
			He constant	Phenotyping	**10.7** (0.3)	**3.4** (0.3)	**2.7** (0.3)	**2.3** (0.3)	**80.5** (6.2)	**68.5** (10.4)	**21.1** (2.0)	**19.4** (0.8)	**13.5** (0.5)	**11.7** (0.5)	**10.4** (0.4)	**86.7** (2.9)	**77.4** (2.8)	**47.6** (1.7)
				H crit. + Pheno.	**30.0** (2.3)	**5.9** (0.8)	**3.9** (0.3)	**2.8** (0.3)	**72.6** (6.5)	**55.1** (7.1)	**22.0** (1.9)	**76.3** (2.6)	**27.6** (1.9)	**18.0** (0.9)	**15.6** (0.9)	**66.2** (2.3)	**57.2** (1.8)	**49.8** (1.6)
	Multiple-stage	Joint TS	He constant	GS Prediction	**36.0** (2.3)	**5.2** (0.7)	**4.0** (0.4)	**3.2** (0.3)	**81.8** (5.6)	**66.4** (6.6)	**20.5** (1.5)	**116.5** (2.9)	**28.8** (1.5)	**18.0** (1.2)	**15.1** (0.9)	**62.3** (2.2)	**52.3** (1.5)	**46.1** (1.5)
			He Linear Loss		**35.3** (0.9)	**3.5** (0.5)	**2.4** (0.3)	**1.0** (0.3)	**77.0** (7.8)	**29.7** (11.2)	**17.9** (2.0)	**106.9** (2.9)	**10.8** (0.9)	**7.0** (0.4)	**6.4** (0.6)	**67.4** (4.8)	**61.0** (5.6)	**36.5** (3.1)
			He constant	Phenotyping	**9.7** (0.7)	**4.2** (0.4)	**3.3** (0.3)	**2.7** (0.2)	**79.8** (4.9)	**67.8** (5.5)	**19.9** (2.6)	**22.2** (1.1)	**15.0** (0.8)	**12.6** (0.7)	**11.5** (0.7)	**84.5** (3.5)	**76.8** (2.9)	**47.7** (2.0)
				H crit. + Pheno.	**24.9** (1.5)	**5.9** (0.4)	**4.4** (0.3)	**3.7** (0.4)	**75.1** (4.6)	**61.9** (3.8)	**22.9** (2.3)	**77.2** (2.3)	**25.9** (1.7)	**17.4** (1.4)	**15.0** (1.1)	**67.0** (2.5)	**58.3** (3.0)	**45.2** (1.7)
	Multiple-stage PreSel_DE	Joint TS	He constant	GS Prediction	**35.2** (1.3)	**4.7** (0.5)	**3.6** (0.4)	**2.6** (0.2)	**77.5** (4.8)	**58.9** (4.6)	**20.5** (1.7)	**115.7** (2.2)	**28.3** (2.3)	**18.0** (1.9)	**14.9** (1.7)	**63.2** (3.4)	**52.4** (3.5)	**47.6** (1.6)
			He Linear Loss		**33.4** (1.0)	**3.2** (0.5)	**2.3** (0.3)	**1.8** (0.4)	**75.5** (7.6)	**51.0** (13.0)	**19.1** (2.6)	**106.6** (2.9)	**12.7** (0.8)	**7.8** (0.7)	**7.2** (0.6)	**61.0** (3.0)	**56.7** (3.1)	**39.3** (3.5)
			He constant	Phenotyping	**9.7** (0.6)	**3.7** (0.3)	**3.2** (0.3)	**1.6** (0.5)	**87.2** (5.8)	**42.3** (12.2)	**21.6** (1.7)	**20.1** (1.2)	**13.7** (0.5)	**11.4** (0.4)	**10.4** (0.4)	**83.7** (2.9)	**76.3** (2.4)	**47.4** (2.3)
				H crit. + Pheno.	**24.7** (0.9)	**5.9** (0.4)	**4.2** (0.3)	**3.7** (0.3)	**72.6** (5.2)	**64.8** (6.6)	**21.2** (2.4)	**76.1** (2.0)	**26.2** (1.5)	**17.9** (1.1)	**16.0** (1.2)	**68.6** (2.5)	**61.5** (3.5)	**47.2** (1.0)
$15C_E + 10C_B$	Bridging	Joint TS	He constant	GS Prediction	**60.1** (1.9)	**6.0** (0.8)	**3.6** (0.4)	**2.5** (0.3)	**63.2** (5.3)	**46.4** (8.7)	**21.9** (2.0)	**156.8** (2.7)	**32.2** (2.0)	**18.6** (1.3)	**15.7** (1.0)	**57.9** (2.2)	**49.0** (1.8)	**51.1** (1.8)
				Phenotyping	**15.5** (0.9)	**4.8** (0.4)	**3.5** (0.3)	**2.6** (0.4)	**74.3** (4.0)	**51.7** (7.2)	**24.5** (1.3)	**26.3** (1.3)	**17.9** (0.8)	**14.1** (0.8)	**13.0** (0.6)	**79.1** (3.6)	**73.1** (2.9)	**49.4** (1.6)
				H crit. + Pheno.	**45.4** (2.9)	**7.0** (0.9)	**4.3** (0.5)	**3.3** (0.4)	**67.0** (7.1)	**48.5** (8.6)	**22.8** (1.2)	**104.8** (2.5)	**32.8** (1.9)	**20.1** (1.2)	**16.9** (0.9)	**61.5** (2.3)	**51.8** (1.5)	**53.4** (1.7)
	Multiple-stage	Joint TS	He constant	GS Prediction	**53.3** (1.0)	**5.5** (0.9)	**3.7** (0.4)	**2.5** (0.4)	**74.6** (7.2)	**52.0** (10.3)	**20.9** (2.1)	**154.8** (2.8)	**30.9** (1.8)	**17.9** (1.0)	**15.9** (1.0)	**58.8** (3.4)	**52.3** (3.5)	**48.4** (1.8)
				Phenotyping	**15.6** (0.8)	**4.8** (0.5)	**3.3** (0.3)	**2.3** (0.4)	**71.9** (5.7)	**53.4** (11.2)	**22.2** (2.0)	**31.1** (1.0)	**19.3** (0.7)	**14.9** (0.8)	**12.5** (0.6)	**77.2** (2.4)	**64.9** (2.1)	**47.2** (2.0)
				H crit. + Pheno.	**39.6** (1.6)	**6.5** (0.7)	**4.7** (0.5)	**3.8** (0.4)	**75.3** (4.6)	**61.7** (5.5)	**23.1** (2.3)	**112.1** (2.4)	**29.9** (1.9)	**20.3** (1.0)	**16.7** (0.7)	**68.6** (2.2)	**57.1** (3.0)	**45.5** (2.3)
	Multiple-stage PreSel_DE	Joint TS	He constant	GS Prediction	**51.5** (1.6)	**5.6** (0.7)	**4.0** (0.5)	**3.0** (0.5)	**71.3** (5.1)	**54.5** (8.8)	**21.4** (1.6)	**147.7** (1.7)	**34.8** (2.0)	**19.9** (1.5)	**16.5** (1.4)	**56.9** (1.9)	**47.0** (2.2)	**46.8** (1.3)
				Phenotyping	**16.3** (1.2)	**4.6** (0.4)	**3.5** (0.3)	**3.0** (0.2)	**78.0** (5.2)	**69.8** (7.5)	**23.3** (1.6)	**31.7** (1.1)	**19.7** (1.0)	**15.7** (0.9)	**13.2** (0.7)	**79.6** (2.3)	**67.4** (2.2)	**49.9** (0.9)
				H crit. + Pheno.	**40.5** (2.1)	**6.8** (0.5)	**4.6** (0.4)	**3.5** (0.4)	**69.5** (5.7)	**53.6** (7.1)	**22.7** (1.9)	**108.6** (1.9)	**31.2** (1.5)	**19.6** (1.1)	**16.9** (1.2)	**63.0** (2.4)	**54.1** (2.6)	**47.9** (1.1)
$10C_E + 10C_B$	Bridging	Joint TS	He constant	GS Prediction	**62.3** (1.5)	**5.4** (1.0)	**3.8** (0.6)	**2.7** (0.4)	**75.9** (5.1)	**52.5** (8.9)	**22.5** (1.3)	**155.5** (3.4)	**30.7** (2.3)	**19.4** (1.4)	**16.4** (1.4)	**63.4** (1.7)	**53.4** (1.9)	**54.2** (0.8)
				Phenotyping	**16.2** (1.1)	**4.5** (0.4)	**3.8** (0.4)	**2.9** (0.3)	**84.8** (4.5)	**68.4** (7.2)	**24.4** (1.5)	**26.3** (1.4)	**17.6** (0.5)	**14.1** (0.4)	**12.0** (0.5)	**80.4** (1.9)	**68.3** (2.1)	**55.7** (1.0)
				H crit. + Pheno.	**53.1** (1.4)	**6.7** (0.6)	**4.8** (0.4)	**4.0** (0.3)	**72.4** (3.4)	**61.6** (3.7)	**25.6** (1.9)	**110.7** (3.3)	**29.4** (2.1)	**19.7** (1.3)	**17.9** (1.3)	**67.5** (1.9)	**61.2** (2.1)	**56.3** (1.2)
	Multiple-stage	Joint TS	He constant	GS Prediction	**57.0** (2.2)	**6.2** (1.2)	**3.6** (0.5)	**2.4** (0.3)	**71.6** (9.3)	**46.6** (10.8)	**23.1** (1.9)	**152.4** (2.7)	**30.4** (2.3)	**19.8** (2.0)	**17.8** (1.9)	**64.4** (3.3)	**57.7** (3.1)	**54.5** (2.1)
				Phenotyping	**16.3** (0.8)	**4.7** (0.3)	**3.6** (0.4)	**3.3** (0.3)	**76.3** (5.1)	**71.0** (4.5)	**24.6** (2.1)	**35.3** (2.3)	**18.3** (1.1)	**15.4** (1.0)	**13.8** (1.0)	**84.5** (3.4)	**75.6** (3.1)	**52.6** (3.1)
				H crit. + Pheno.	**44.4** (1.9)	**6.8** (0.9)	**4.5** (0.4)	**3.0** (0.2)	**70.4** (4.8)	**52.7** (8.4)	**24.8** (1.6)	**118.9** (2.1)	**28.7** (2.1)	**19.6** (1.5)	**17.8** (1.4)	**68.5** (2.0)	**62.6** (2.7)	**48.2** (1.4)
	Multiple-stage PreSel_DE	Joint TS	He constant	GS Prediction	**52.9** (1.8)	**5.8** (0.6)	**3.5** (0.3)	**2.5** (0.3)	**65.6** (7.0)	**47.9** (8.7)	**25.7** (1.8)	**145.0** (3.3)	**31.7** (1.4)	**21.4** (1.0)	**19.1** (1.0)	**67.9** (2.3)	**60.5** (2.5)	**56.0** (0.9)
				Phenotyping	**15.9** (0.8)	**4.8** (0.6)	**3.7** (0.3)	**3.0** (0.3)	**82.5** (6.7)	**68.9** (8.8)	**23.3** (2.4)	**29.0** (0.7)	**18.3** (1.0)	**15.0** (0.8)	**13.2** (0.7)	**82.7** (3.0)	**72.7** (2.6)	**55.0** (2.1)
				H crit. + Pheno.	**44.6** (1.6)	**7.4** (0.6)	**5.4** (0.5)	**4.7** (0.5)	**74.3** (6.4)	**64.7** (6.7)	**24.1** (2.0)	**113.9** (2.8)	**30.3** (2.2)	**20.9** (1.8)	**18.5** (1.4)	**69.1** (3.8)	**62.1** (4.2)	**50.7** (1.2)

N_{SD}: Number of different donors selected as bridging parents until generations 15 or 60. N_{ID}: Number of donors with progenies introduced into elite components until generation 15 or 60. N_{CD}: Number of donors with progenies in Elite at generations 15 or 60. $N_{CDtop10}$: Number of donors with progenies in the top 10 lines of generation 15 or 60. % Elite Genome: Percentage of elite line genome coming from donors, based on pedigree information. H crit. + Pheno: H criterion + Phenotyping. Values between parentheses indicate standard errors of the estimated values.

Table S9 Recycling of DE progenies in the bridging component and the effects of the diversity management

			Number of crosses in the bridging		μ at generation 60	
**Allocation**	**Strategy**	**Donor Selection Strategy**	**He Elite Constant**	**He Breeding Constant + He Elite non Constant**	**He Elite Constant**	**He Breeding Constant + He Elite non Constant**
$15C_E + 5C_B$	Multiple-stage	GS Prediction	**1.03** (0.02)	**3.25** (0.09)	**39.93** (1.24)	**37.82** (1.11)
		Phenotyping	**1.08** (0.02)	**3.29** (0.18)	**40.77** (0.79)	**38.57** (1.03)
		H criterion + Phenotyping	**1.29** (0.03)	**3.21** (0.12)	**41.08** (1.30)	**37.81** (0.95)
	Multiple-stage PreSel_DE	GS Prediction	**1.01** (0.01)	**2.87** (0.11)	**40.97** (1.41)	**37.24** (1.17)
		Phenotyping	**1.05** (0.01)	**3.08** (0.18)	**39.98** (0.96)	**38.30** (1.09)
		H criterion + Phenotyping	**1.18** (0.03)	**3.00** (0.18)	**40.49** (1.24)	**39.33** (1.53)
$15C_E + 10C_B$	Multiple-stage	GS Prediction	**1.08** (0.03)	**3.42** (0.17)	**39.15** (1.07)	**38.72** (1.23)
		Phenotyping	**1.14** (0.05)	**3.70** (0.13)	**39.94** (1.24)	**38.40** (1.36)
		H criterion + Phenotyping	**1.50** (0.05)	**3.94** (0.25)	**40.60** (1.01)	**39.11** (1.50)
	Multiple-stage PreSel_DE	GS Prediction	**1.08** (0.02)	**3.17** (0.15)	**40.11** (1.11)	**39.03** (1.13)
		Phenotyping	**1.11** (0.02)	**3.58** (0.20)	**40.42** (1.40)	**39.37** (1.07)
		H criterion + Phenotyping	**1.34** (0.05)	**3.38** (0.22)	**40.63** (0.96)	**39.62** (1.28)
$10C_E + 10C_B$	Multiple-stage	GS Prediction	**1.10** (0.04)	**4.22** (0.31)	**38.44** (1.17)	**37.52** (1.26)
		Phenotyping	**1.18** (0.04)	**4.17** (0.18)	**40.37** (1.66)	**39.13** (1.57)
		H criterion + Phenotyping	**1.56** (0.05)	**4.06** (0.15)	**39.66** (1.03)	**39.70** (1.51)
	Multiple-stage PreSel_DE	GS Prediction	**1.09** (0.04)	**3.51** (0.14)	**39.10** (1.02)	**38.79** (1.34)
		Phenotyping	**1.10** (0.03)	**3.68** (0.17)	**38.86** (1.08)	**39.27** (1.39)
		H criterion + Phenotyping	**1.28** (0.03)	**3.76** (0.12)	**39.33** (0.70)	**40.84** (1.27)

Number of crosses in the bridging: number of times a donor is crossed with elite lines in the bridging before its progeny is incorporated into the elite. "He Breeding Constant + He Elite non Constant" is an alternative diversity management: the diversity at neutral markers is maintained constant in the whole breeding component (elite + bridging) and the elite diversity is allowed to decrease until reaching half of the initial diversity observed at the end of burn-in phase. Values between parentheses indicate standard errors of the estimated values.

SI References

1. A. Allier, S. Teyssedre, C. Lehermeier, A. Charcosset, L. Moreau, Genomic prediction with a maize collaborative panel: identification of genetic resources to enrich elite breeding programs. THEORETICAL AND APPLIED GENETICS 133, 201–215 (2020).

2. A. Allier, C. Lehermeier, A. Charcosset, L. Moreau, S. Teyssèdre, Improving Short- and Long-Term Genetic Gain by Accounting for Within-Family Variance in Optimal Cross-Selection. Frontiers in Genetics 10 (2019).

3. J. a. Woolliams, P. Berg, B. s. Dagnachew, T. h. e. Meuwissen, Genetic contributions and their optimization. Journal of Animal Breeding and Genetics 132, 89–99 (2015).

4. A. Allier, S. Teyssèdre, C. Lehermeier, L. Moreau, A. Charcosset, Optimized breeding strategies to harness genetic resources with different performance levels. BMC Genomics 21, 349 (2020).
